# Supplementary material for: Iridium metallene oxide for acidic oxygen evolution catalysis
Source: Nat Commun. 2021 Oct 14;12:6007. doi: 10.1038/s41467-021-26336-2 (PMC8516950; doi:10.1038/s41467-021-26336-2)
Supplement: Supplementary file 1 — Supplementary Information [file 41467_2021_26336_MOESM1_ESM.pdf]

Supplementary Figures

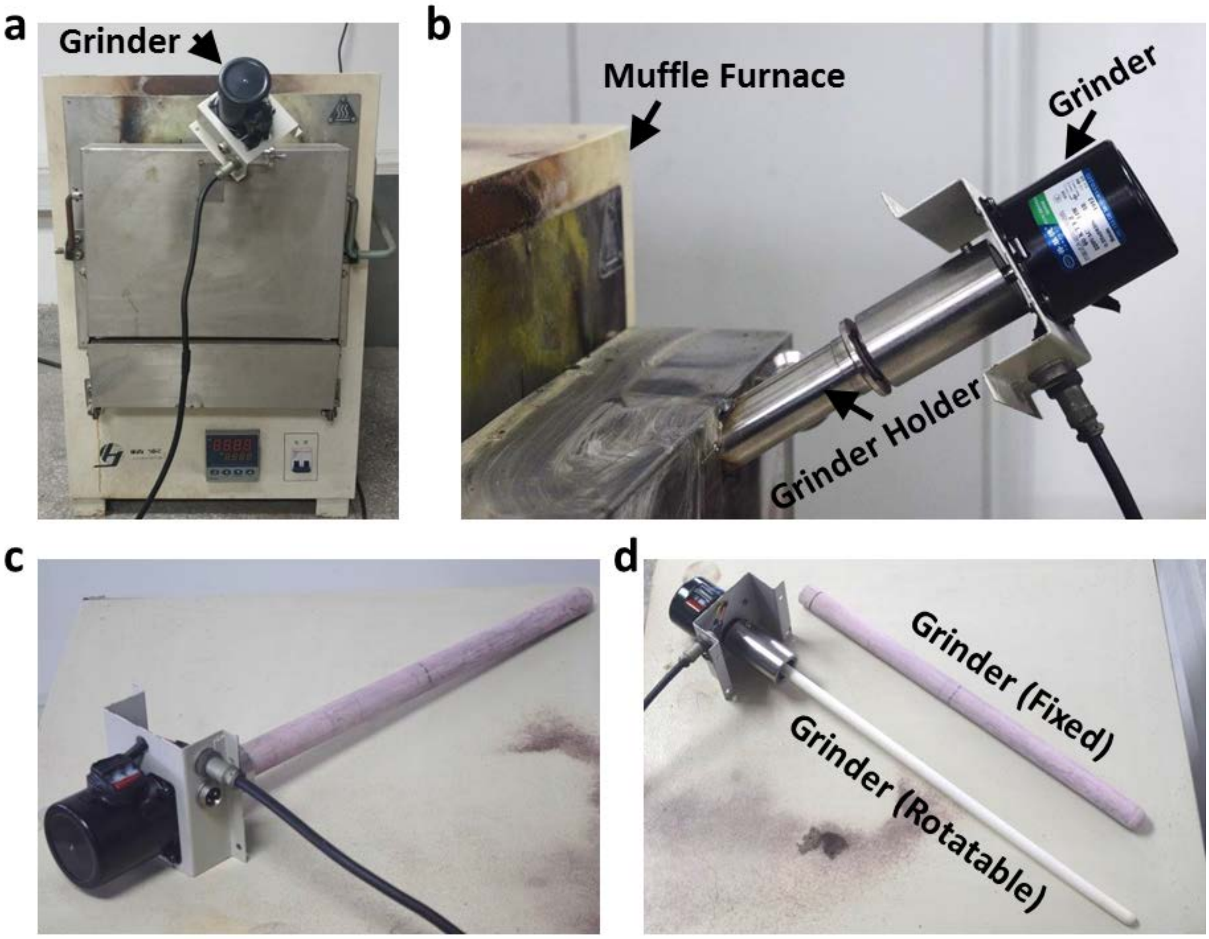

Supplementary Figure 1. The digital images of a, the home-made mechano-thermal reactor for the synthesis of 1T-IrO_2 and b, the mechano-thermal reactor. The digital images of c, grinder and d, its exploded picture.

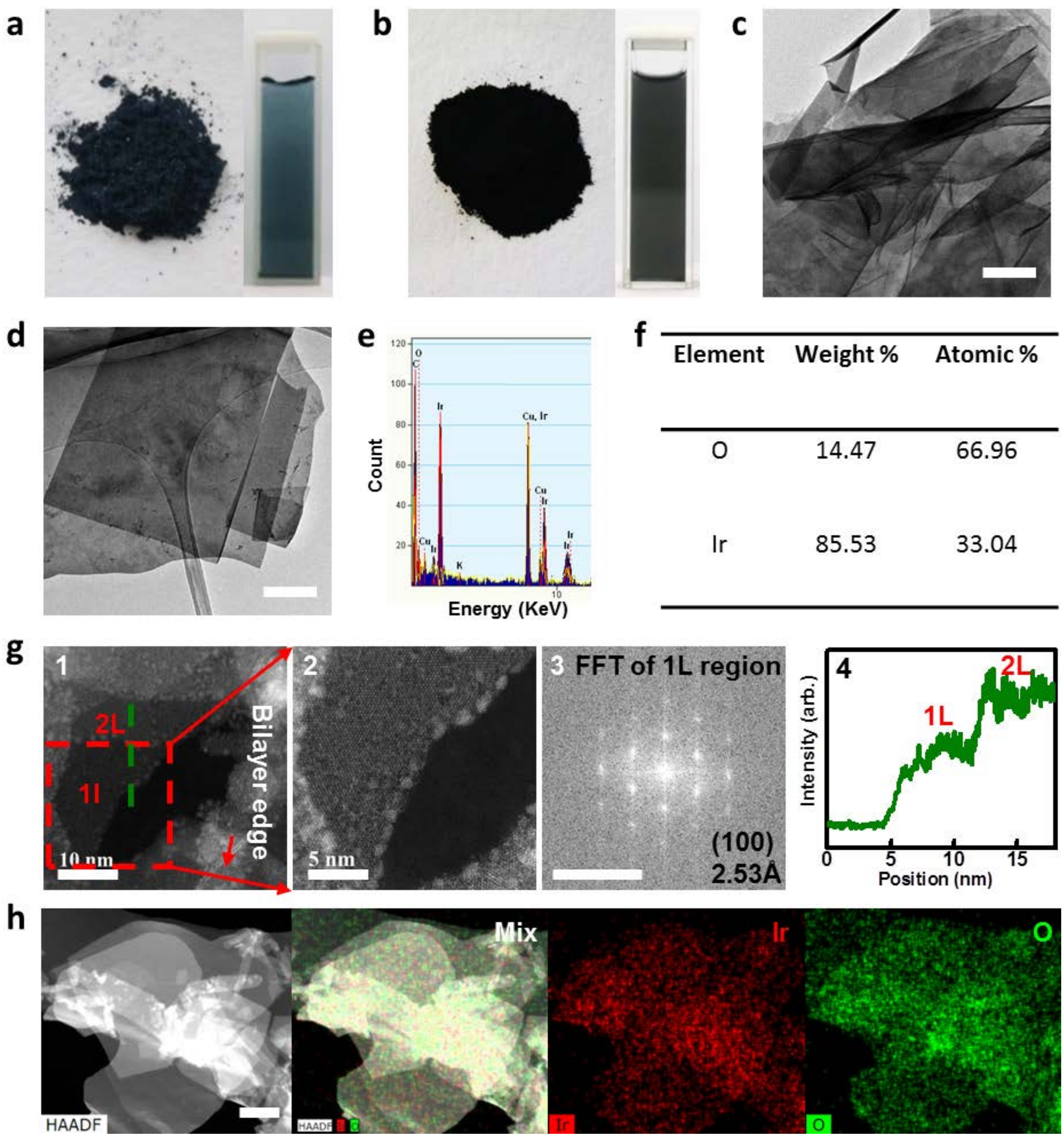

Supplementary Figure 2. The digital images of a, 1T-IrO_2 powder and a inset, its water dispersion in a cuvette. The digital images of b, rutile-IrO_2 powder and b inset, its water dispersion in a cuvette. c,d, Transmission electron microscopy (TEM) images of 1T-IrO_2, showing the sheet morphology. e, TEM energy-dispersive X-ray spectroscopy (TEM-EDX) result and f, table of the weight and atomic ratios of Ir to O in 1T-IrO_2, revealing the atomic ratio of Ir/O is about 1 : 2. g1, A linear annular dark-field scanning transmission electron microscopy (STEM-ADF) image showing a region with different thicknesses (1L and 2L). The bilayer edge is visible, which is a clear evidence of the two-dimensional structure. An enlarged region for the 1L is shown in g2 and the fast fourier transform (FFT) of the 1L region is shown in g3. g4, The intensity profile across the green line in g1. The nearly doubled intensity of the 2L vs the 1L region is also typical for two-dimensional materials. h, STEM-EDX mapping of 1T-IrO_2, where Ir and O are uniformly distributed. The scale bars in (c), (d), (g3) and (h) are 200 nm, 200 nm, 10 1/nm and 100 nm, respectively.

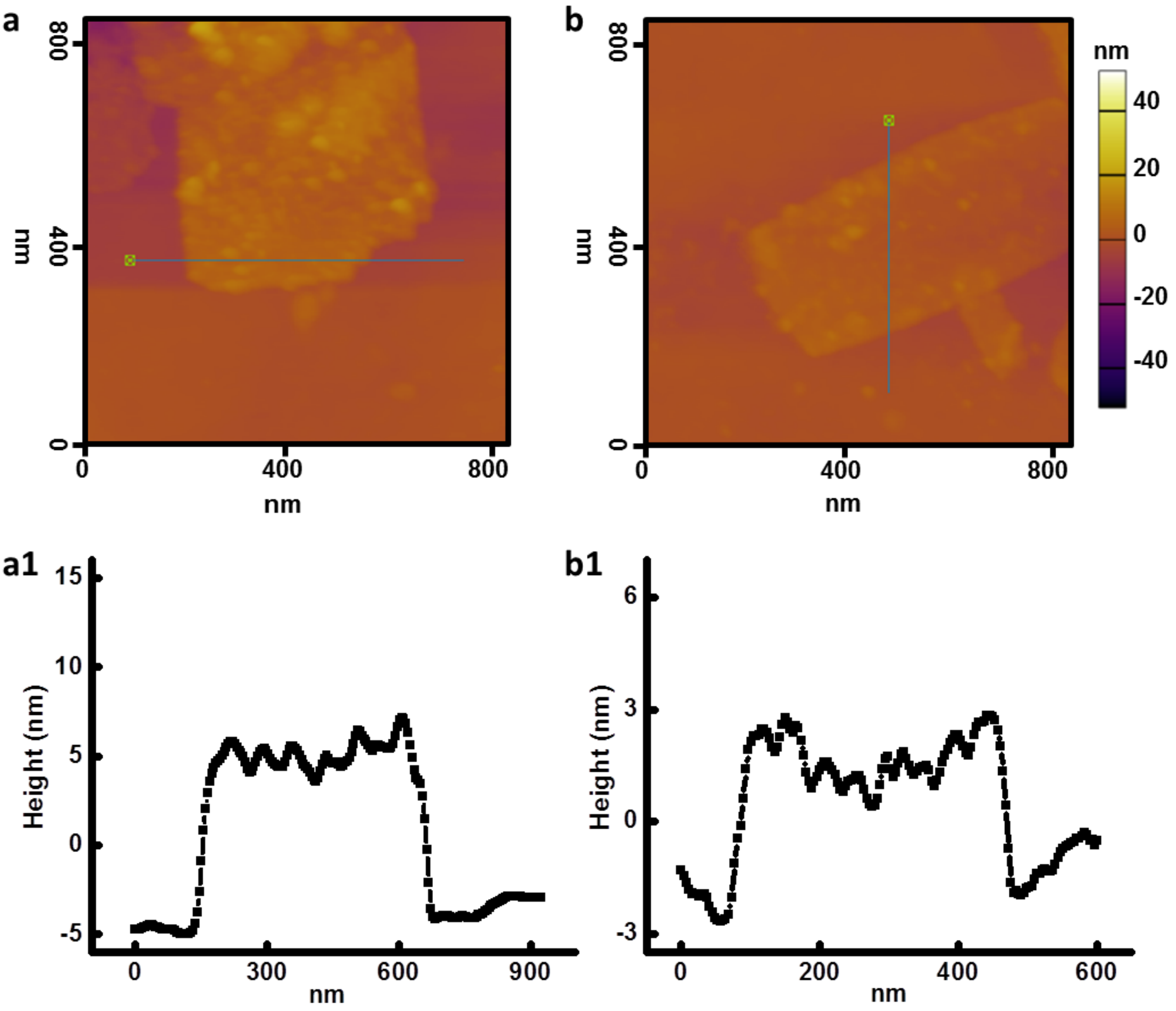

Supplementary Figure 3. a,b, Atomic force microscopy (AFM) images and a1,b1, corresponding height profiles of 1T-IrO_2, revealing its ultrathin morphology.

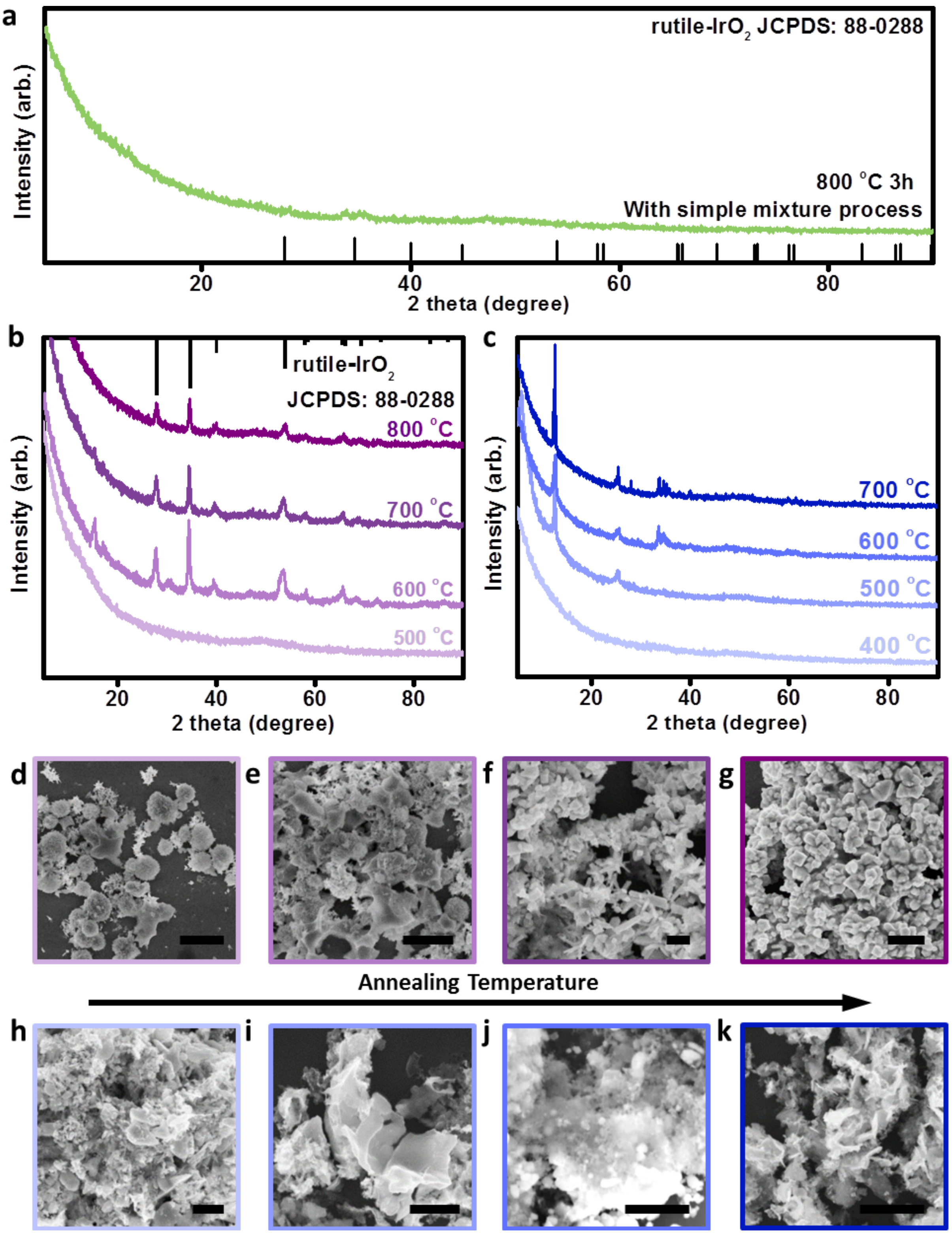

Supplementary Figure 4. a, X-ray diffraction (XRD) pattern of the product by annealing the compound of $IrCl_3$ and KOH, which is obtained by a simple mixture process. b, Temperature-dependent XRD patterns of annealing $IrCl_3$ in air. The annealing temperature is from 500 °C to 800 °C. d-g, The corresponding scanning electron microscope (SEM) images of the resulting powders annealing at (d) 500 °C, (e) 600 °C, (f) 700 °C and (g) 800 °C, indicating the important role of KOH in forming the 1T structure. c, Temperature-dependent XRD patterns of the mechanical mixture of $IrCl_3$ and KOH. The annealing temperature is from 400 °C to 700 °C. h-k, The corresponding SEM

images of the resulting products annealing at (h) 400 °C, (i) 500 °C, (j) 600 °C and (k) 700 °C, suggesting that high temperature annealing treatment is vital for forming high crystalline. The scale bars in (d), (e), (f) and (g) are 1 μm, 1 μm, 200 nm and 500 nm, respectively. The scale bars in (h), (i), (j) and (k) are all 1 μm.

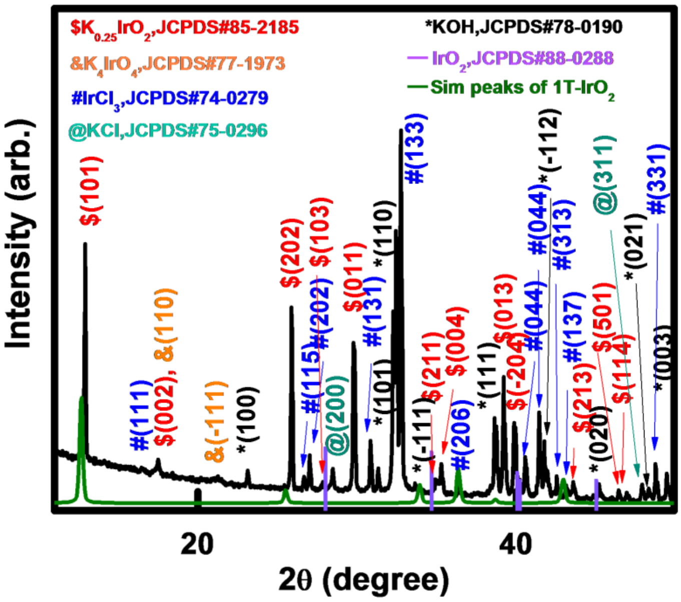

Supplementary Figure 5. XRD pattern of the product by annealing $IrCl_3$ and KOH via the mechano-thermal process, where the annealing temperature is 200 °C. The reaction intermediates of $K_{0.25}IrO_2$ and K_4IrO_4 were detected. The sample for XRD testing was not washed by double-distilled water.

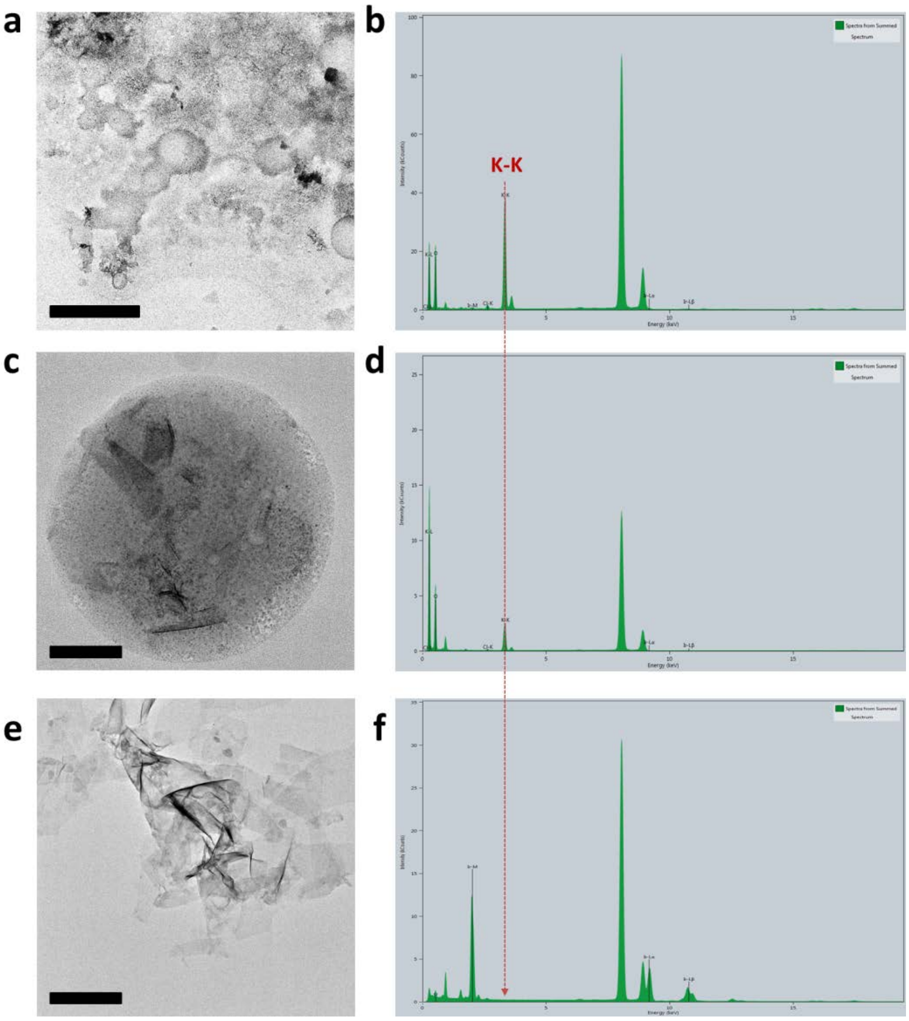

Supplementary Figure 6. The TEM images and TEM-EDX analyses of (a, b) the product by annealing $IrCl_3$ and KOH via the mechano-thermal process without double-distilled water washing, where the annealing temperature is 200 °C, (c, d) the final product of 1T-IrO_2 before washing by double-distilled water and (e, f) the final product of 1T-IrO_2 after washing by double-distilled water. The results reveal that no K element can be found in the final product of 1T-IrO_2 after washing. The scale bars in (a), (c) and (e) are 500 nm, 100 nm and 500 nm.

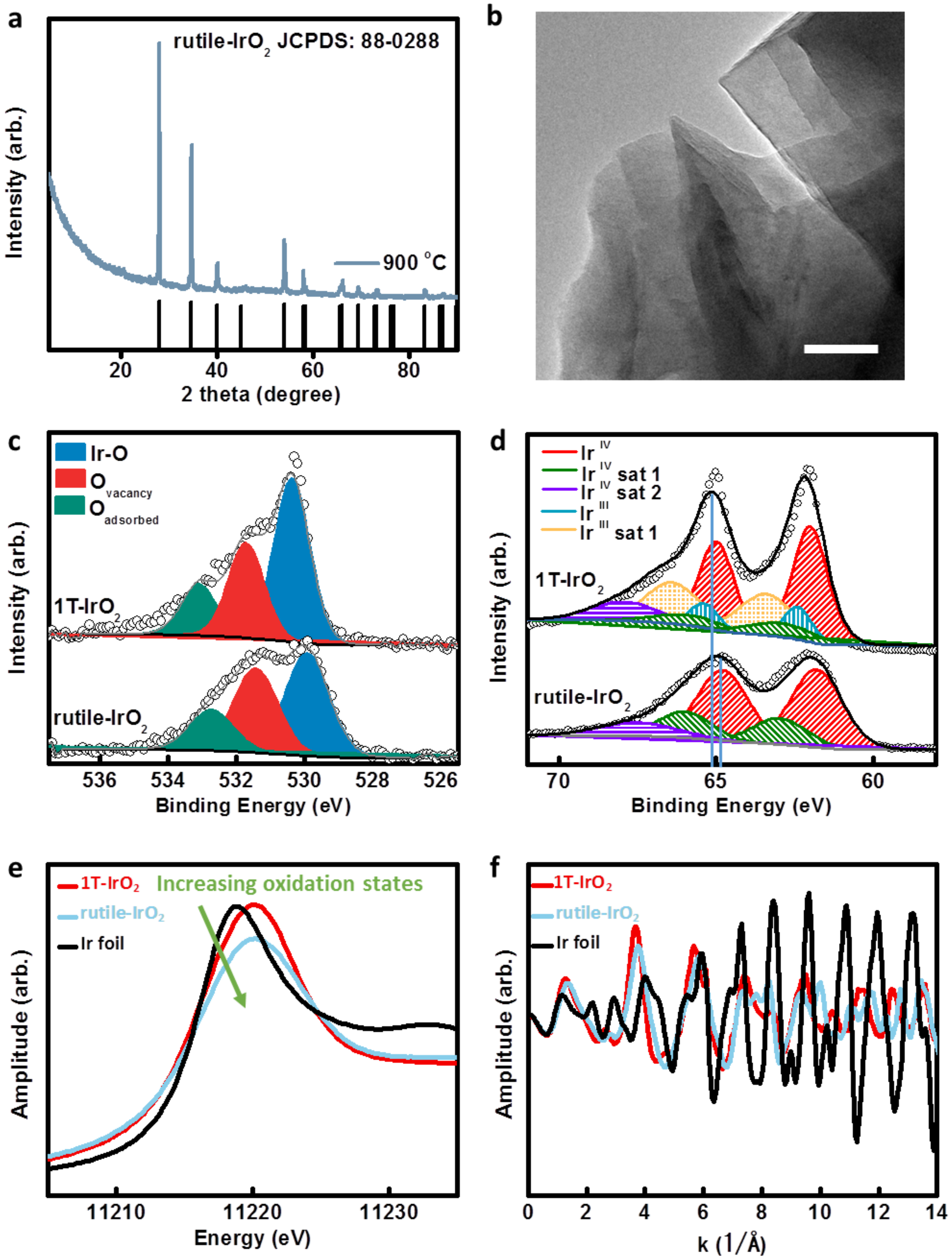

Supplementary Figure 7. a, XRD pattern of Rutile-IrO_2, obtained by annealing 1T-IrO_2 at 900 °C for 2 h. b, The corresponding TEM image of Rutile-IrO_2, showing its sheet morphology. c, X-ray photoelectron spectroscopy (XPS) spectra of O 1*s* peaks for 1T-IrO_2 and rutile-IrO_2. O 1*s* peak presents many spectral contributions that were successfully disentangled by fitting analysis, resulting in three nearly Gaussian components for Ir-O lattice bond, oxygen vacancy and surface chemisorbed or dissociated oxygen or OH species, respectively. d, XPS spectra of Ir 4*f*

peaks for 1T-IrO_2 and rutile-IrO_2. The Ir 4*f* peak in 1T-IrO_2 shifts to the higher binding energy compared to those in rutile-IrO_2, suggesting that Ir^{3+} species are existing on the surface of 1T-IrO_2. e, Enlarged region from Fig. 3a to show the change in oxidation valence state of Ir, suggesting more metallic state on 1T-IrO_2. f, k-space view of the extended X-ray absorption fine structure (EXAFS) data for 1T-IrO_2, rutile-IrO_2 and Ir foil. The scale bar in (b) is 50 nm.

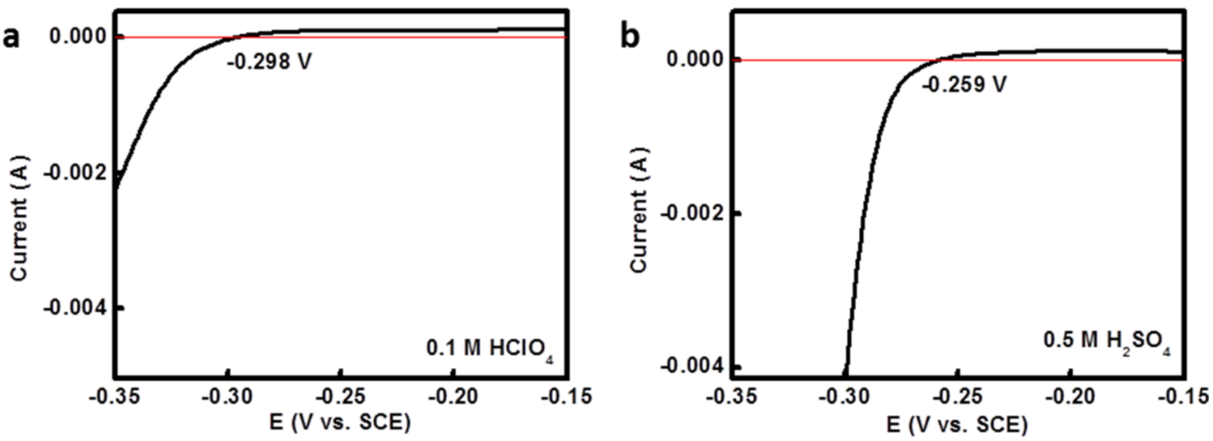

Supplementary Figure 8. Calibration of the saturated calomel electrode (SCE) electrode with respect to reversible hydrogen electrode (RHE) in a, 0.1 M $HClO_4$ and b, 0.5 M H_2SO_4 aqueous electrolytes bubbled with pure hydrogen gas at room temperature. Scan rate: 1 mV s^{-1}.

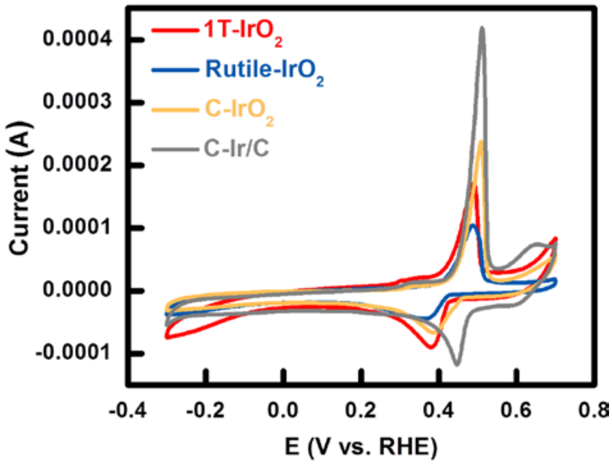

Supplementary Figure 9. Mercury underpotential deposition method was used to determine the surface area. CV curves of 1T-IrO_2, Rutile-IrO_2, C-IrO_2 and C-Ir/C in 0.1 M $HClO_4$ containing 1.0 mM mercury nitrate. The scan rate is 100 mV s^{-1}.

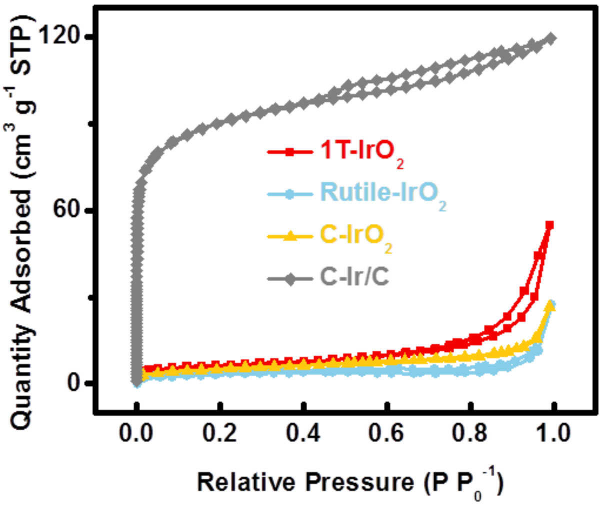

Supplementary Figure 10. Brunauer-Emmett-Teller (BET) surface area measurements for 1T-IrO_2, Rutile-IrO_2, C-IrO_2 and C-Ir/C.

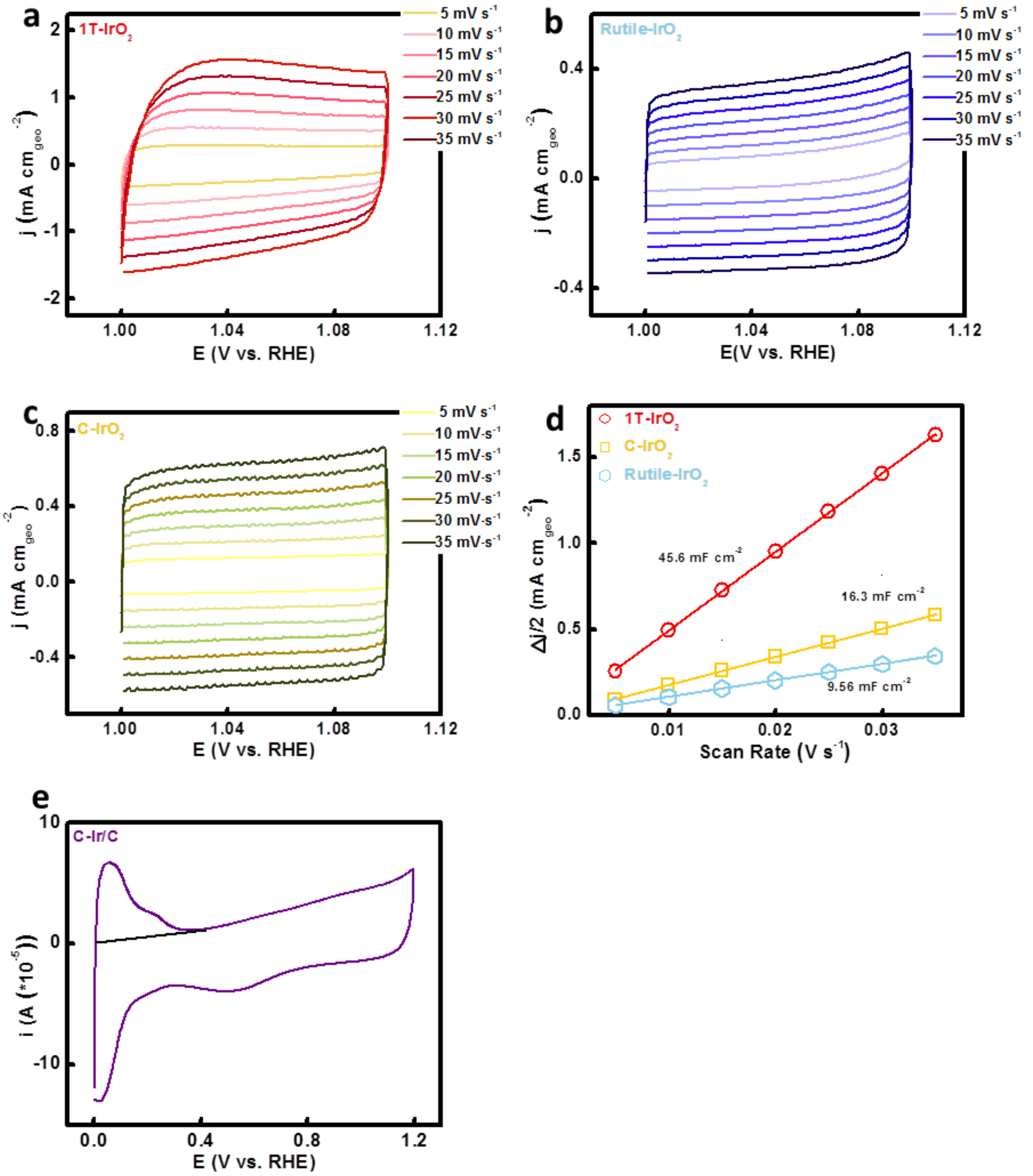

Supplementary Figure 11. CV curves of a, 1T-IrO_2, b, Rutile-IrO_2 and c, C-IrO_2 in double layer region. d, Plots of the charging current density as functions of the scan rate of 1T-IrO_2, Rutile-IrO_2 and C-IrO_2. The scan rate increases from 5 to 35 mV s^{-1} per 5 intervals. e, CV curves recorded from C-Ir/C with the scan rate of 5 mV s^{-1}. The electrolyte used is 0.5 M H_2SO_4.

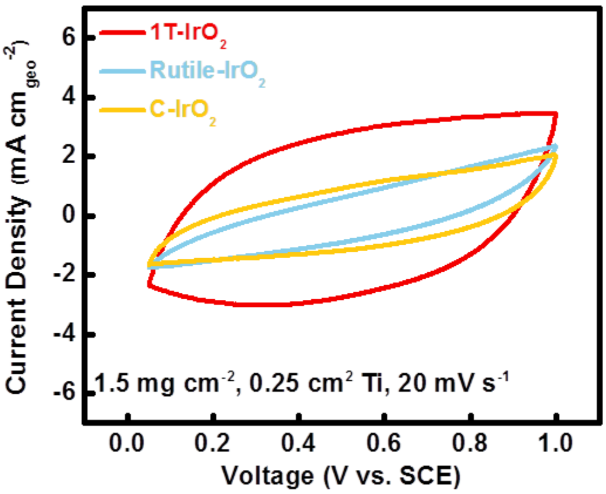

Supplementary Figure 12. Experimental current density-potential curves for 1T-IrO_2, Rutile-IrO_2 and C-IrO_2 coating on titanium (Ti) substrates in 1.0 M H_2SO_4 electrolyte with the scan rate of 20 mV s^{-1}.

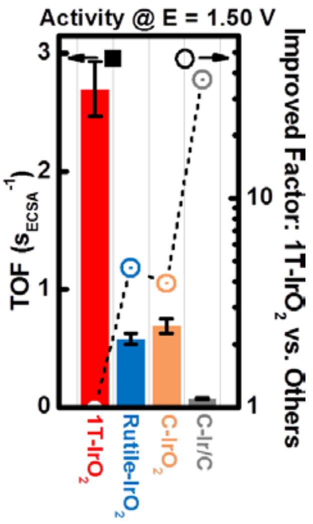

Supplementary Figure 13. Comparison of ECSA-based TOF values at 1.50 V vs. RHE for 1T-IrO_2, Rutile-IrO_2, C-IrO_2 and C-Ir/C in 0.1 M O_2-saturated $HClO_4$ electrolyte with *i*R-correction. Error bars are means ± SD (n = 3 replicates).

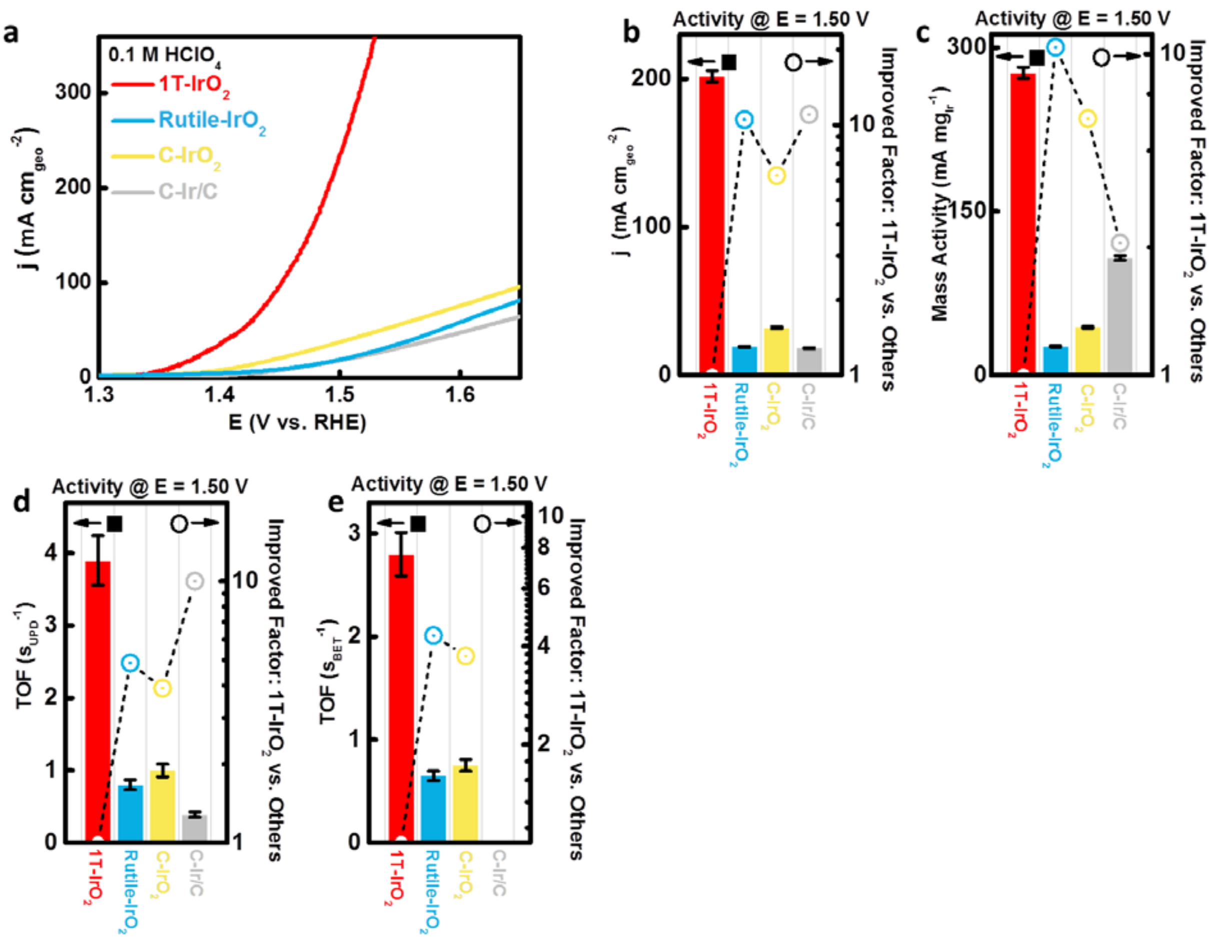

Supplementary Figure 14. a, Polarization curves of 1T-IrO_2, Rutile-IrO_2, C-IrO_2 and C-Ir/C in O_2-saturated 0.1 M $HClO_4$ electrolyte with *i*R-correction. b-e, Comparison of geometric activities, mass activities, UPD-based TOF values and BET-based TOF values at 1.50 V vs. RHE for 1T-IrO_2, Rutile-IrO_2, C-IrO_2 and C-Ir/C. The area of glassy carbon electrode is 1 cm × 1 cm and the mass loading is 0.85 $mg_{catalyst}$. Error bars are means ± SD (n = 3 replicates).

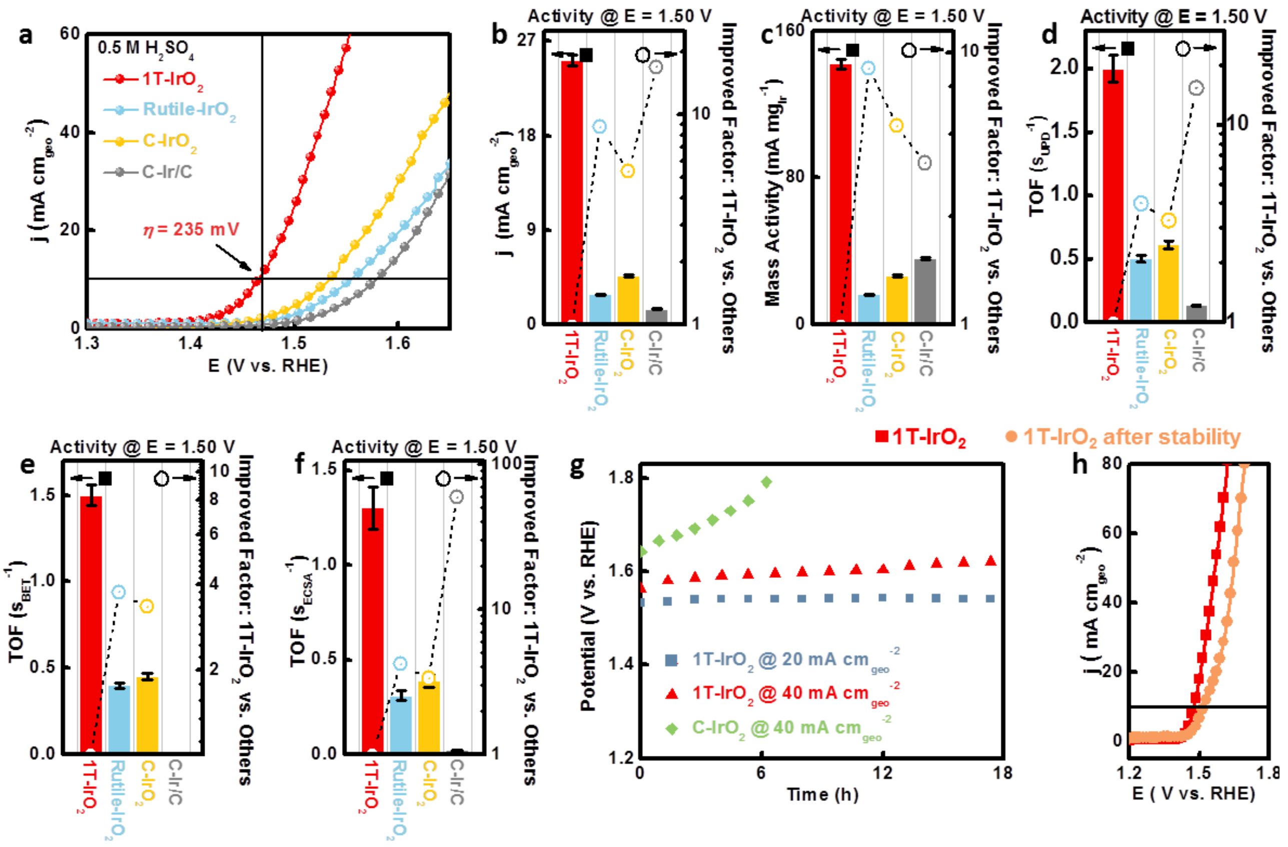

Supplementary Figure 15. a, Polarization curves of 1T-IrO_2, Rutile-IrO_2, C-IrO_2 and C-Ir/C in O_2-saturated 0.5 M H_2SO_4 electrolyte with *i*R-correction, where 1T-IrO_2 deliver an overpotential of 235 mV to achieve the current density of 10 mA cm_{geo}^{-2}. b-f, Comparison of geometric activities, mass activities, UPD-based TOF values, BET-based TOF values, and ECSA-based TOF values at 1.50 V vs. RHE for 1T-IrO_2, Rutile-IrO_2, C-IrO_2 and C-Ir/C. g, Chronopotentiometry performance under different constant current densities for 1T-IrO_2 and C-IrO_2, where 1T-IrO_2 maintain high electrochemical activity even under high current density up to 40 mA cm_{geo}^{-2}. h, The geometric polarization curves of 1T-IrO_2 before and after stability test under the high current density of 40 mA cm_{geo}^{-2} in O_2-saturated 0.5 M H_2SO_4 electrolyte. Error bars are means ± SD (n = 3 replicates).

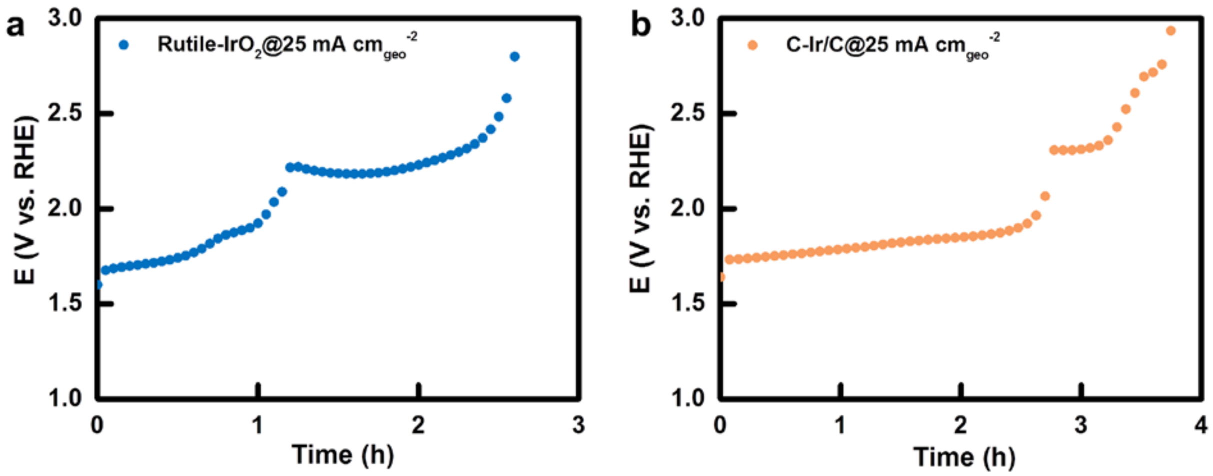

Supplementary Figure 16. Stability tests for Rutile-IrO_2 and C-Ir/C. Chronopotentiometry performances of a, Rutile-IrO_2 and b, C-Ir/C under a constant current density of 25 mA cm_{geo}^{-2}.

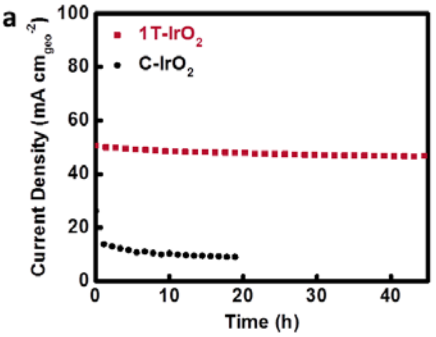

b

ICP-MS results	
**Catalyst**	Dissolved iridium after stability test at 1.53 V vs. RHE for different times (μg_{Ir} L^{-1})
**1T-IrO_2**	6.22 ± 1.1 (after 20 hours)
**1T-IrO_2**	9.28 ± 1.4 (after 45 hours)
**C-IrO_2**	13.45 ± 1.6 (after 20 hours)

Supplementary Figure 17. a, Amperometric test under the high constant voltage of 1.53 V vs. RHE for 1T-IrO_2 and C-IrO_2, where 1T-IrO_2 maintain high electrochemical activity. The working electrolyte is 0.1 M O_2-saturated $HClO_4$. b, ICP-MS results of 1T-IrO_2 and C-IrO_2 after the stability tests. The quantity of leached elements into the electrolyte during OER was investigated via ICP-MS. The ICP-MS results were measured at least three times for each catalyst.

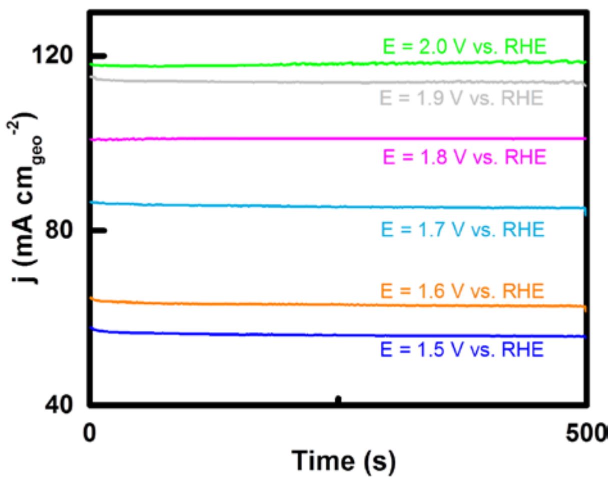

Supplementary Figure 18. The stability tests of 1T-IrO_2 under different potentials (vs. RHE).

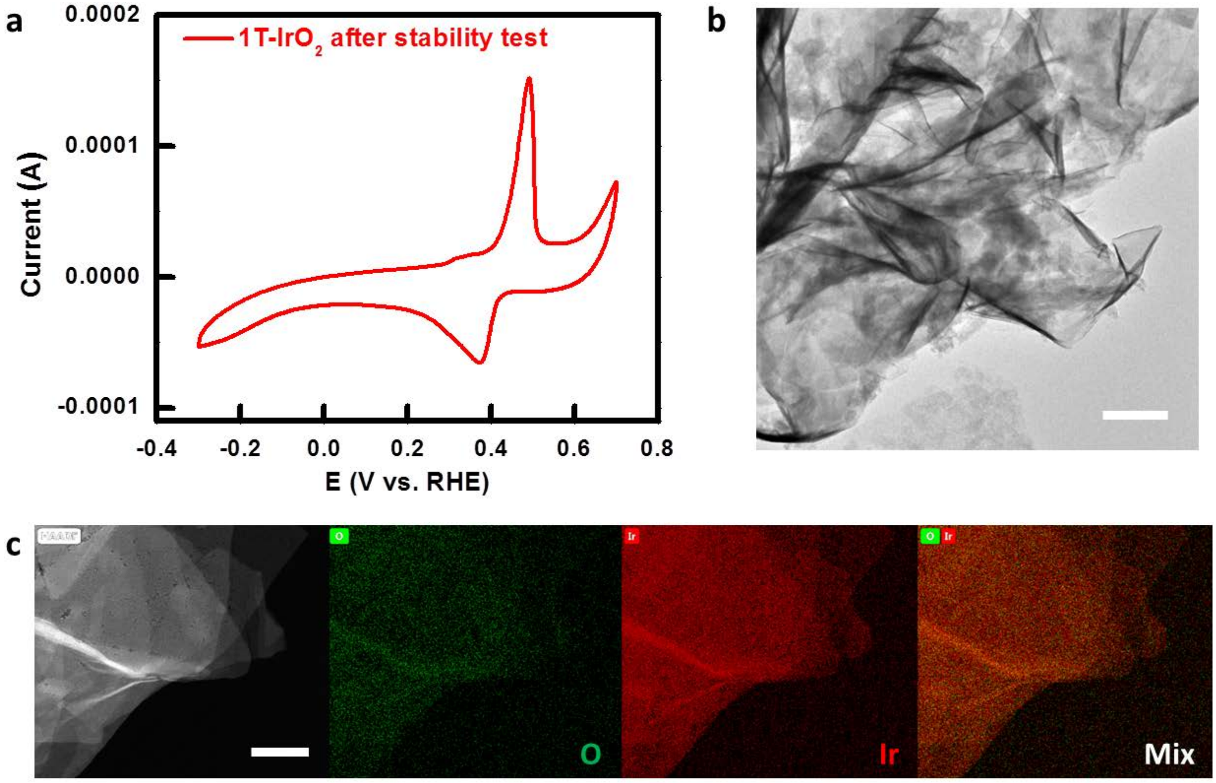

Supplementary Figure 19. a, Mercury underpotential deposition method was used to determine the surface area of 1T-IrO_2 after stability test. CV curve of 1T-IrO_2 after the stability test. The electrolyte used is 0.1 M $HClO_4$ containing 1.0 mM mercury nitrate. b, The TEM image of 1T-IrO_2 after stability test with the current density of 50 mA cm_{geo}^{-2}, clearly showing its sheet morphology. c, TEM-EDX mapping of 1T-IrO_2 after stability test, where Ir and O are uniformly distributed. The scale bars in (b) and (c) are 200 nm and 100 nm.

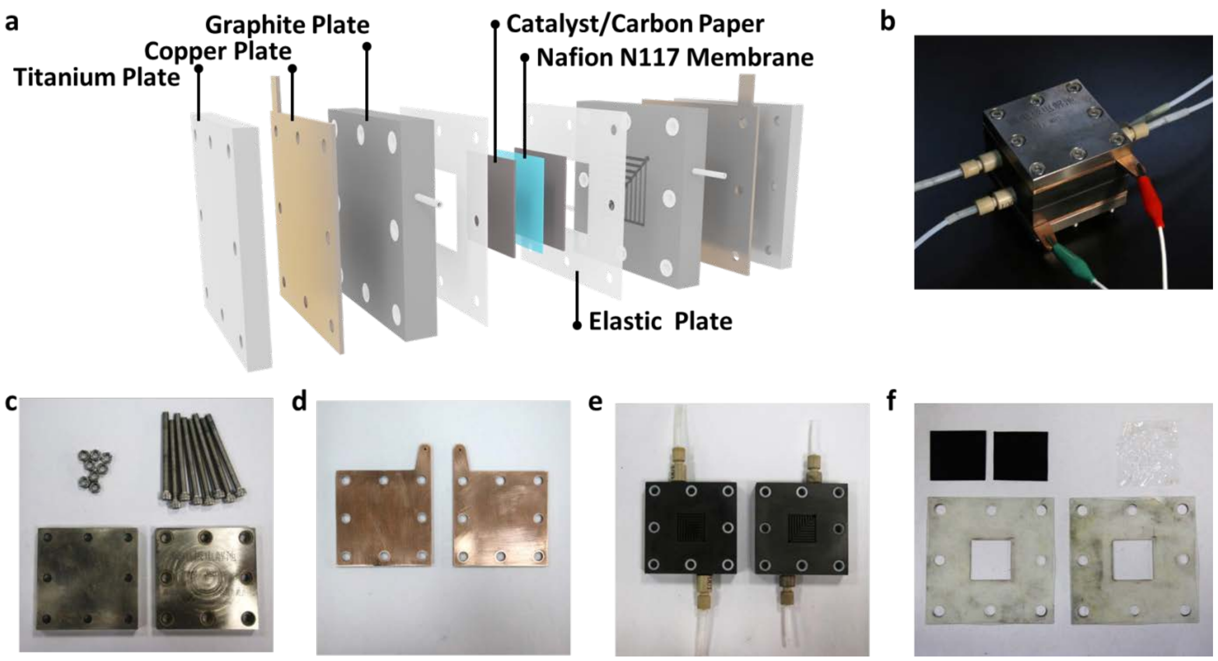

Supplementary Figure 20. a, Schematic illustration of the PEM device. b-f, Photograph of the employed PEM device for acidic water electrolysis.

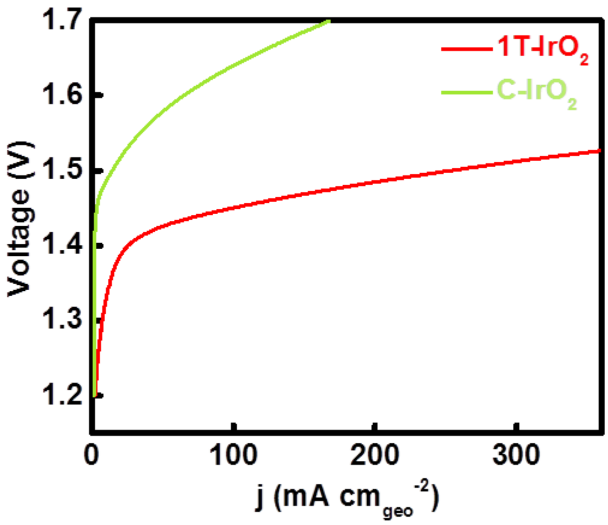

Supplementary Figure 21. Polarization curves of 1T-IrO_2 and C-IrO_2 in O_2-saturated 0.5 M H_2SO_4 electrolyte in PEM device without *i*R-correction. The mass loading of 1T-IrO_2 or C-IrO_2 is 0.85 $mg_{catalyst}$. The reaction temperature is 65 °C.

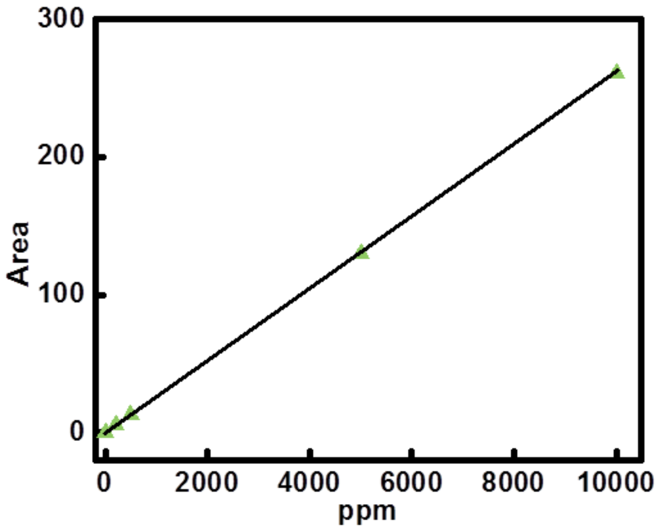

Supplementary Figure 22. Calibration curve of the production of O_2 in the gas chromatograph.

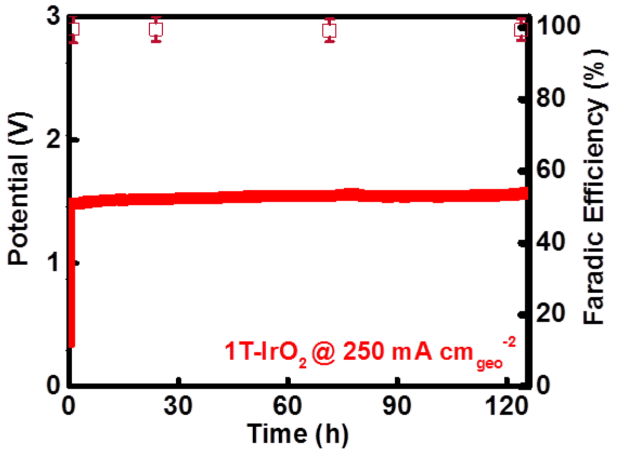

Supplementary Figure 23. Chronopotentiometry performance and Faradic efficiencies of 1T-IrO_2 at the current density of 250 mA cm_{geo}^{-2} in PEM device with using 0.5 M H_2SO_4 at 65 °C. Nafion N117 membrane was used for PEM device. Error bars are means ± SD (n = 3 replicates).

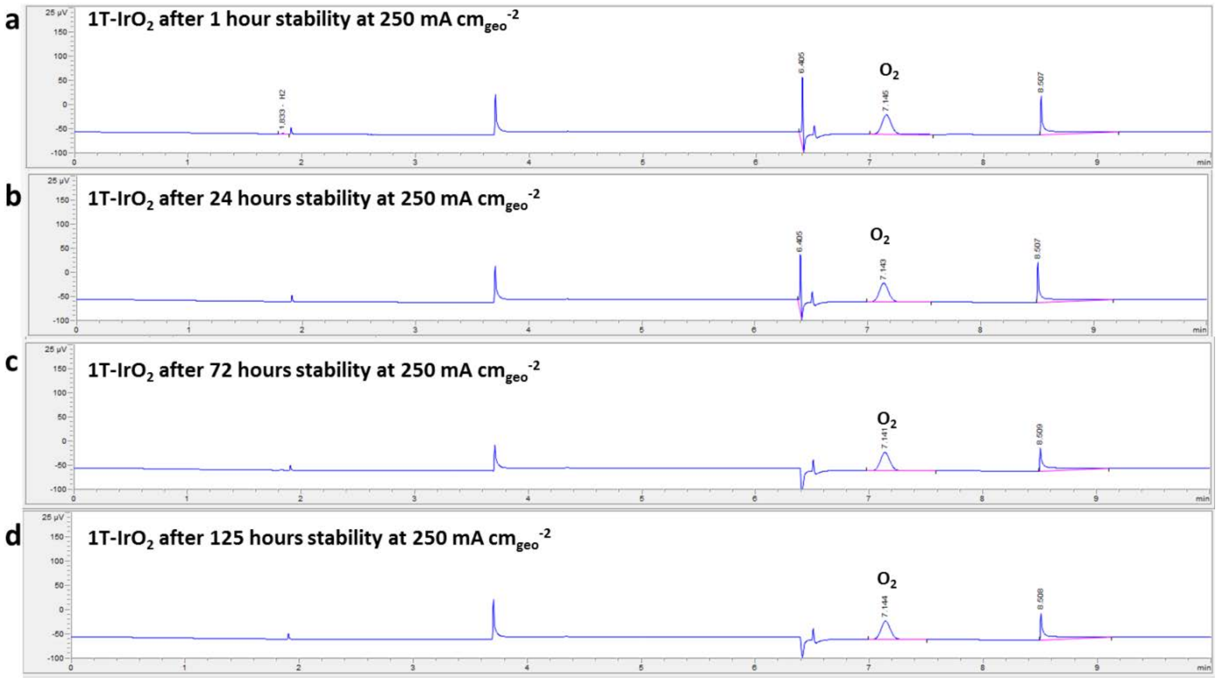

Supplementary Figure 24. GC data of the produced oxygen by 1T-IrO$_2$ at the current density of 250 mA cm_{geo}^{-2} in PEM device after a, 1 hour stability, b, 24 hours stability, c, 72 hours stability and d, 125 hours stability tests. The working electrolyte is 0.5 M O_2-saturated H_2SO_4. The reaction temperature is 65 °C. The produced oxygen was measured at least three times for each catalyst. The flow rate of carrier gas Ar is 100 sccm.

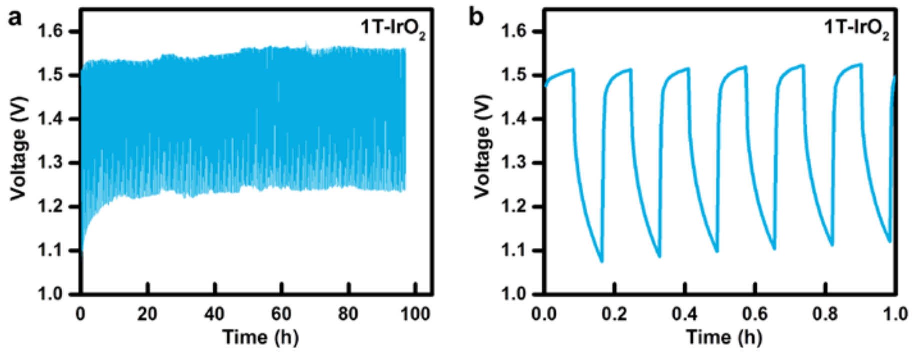

Supplementary Figure 25. a, The start-stop cycles stability test of 1T-IrO_2 and b, the start-stop cycles stability test in the first one hour.

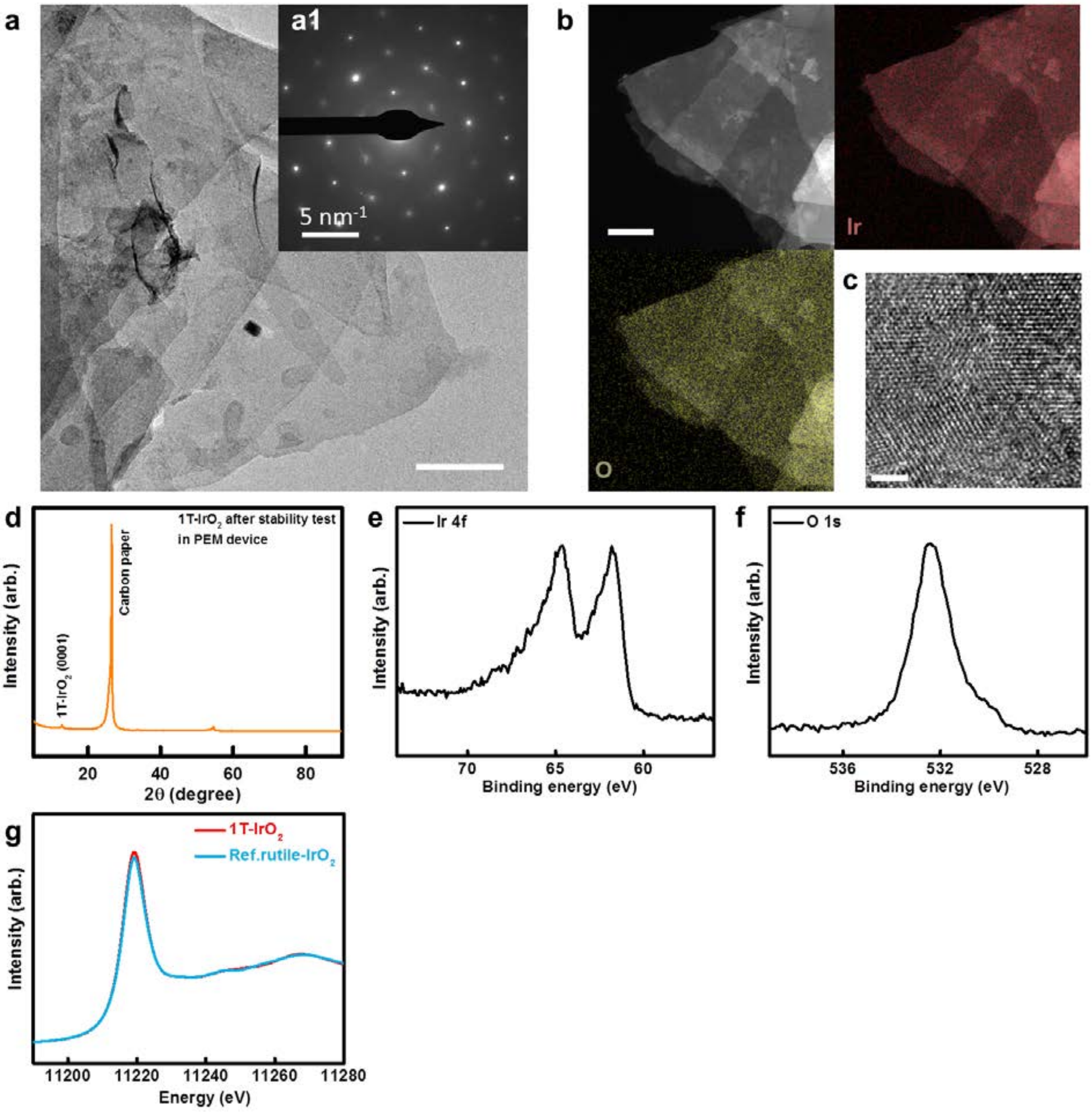

Supplementary Figure 26. a, The TEM image of 1T-IrO_2 after stability test in PEM device with the current density of 250 mA cm_{geo}^{-2}, clearly showing its sheet morphology. a1, The SAED pattern of 1T-IrO_2 in Supplementary Fig. 26a, showing the six-fold rotational symmetry. b, TEM-EDX mapping of 1T-IrO_2 after stability test in PEM device, where Ir and O are uniformly distributed. c, The HRTEM image of 1T-IrO_2 after the OER stability test. The scale bars in (a), (b) and (c) are 100 nm, 100 nm and 2 nm. d, XRD pattern of 1T-IrO_2 after stability test in PEM device. The XRD diffraction peak at 12.81° corresponds to the (0001) peak of 1T-IrO_2. XPS spectra of e, Ir 4*f* and f, O 1*s* peaks for 1T-IrO_2 after stability test in PEM device. g, X-ray absorption near-edge spectroscopy spectra of 1T-IrO_2 after stability test and Reference rutile-IrO_2.

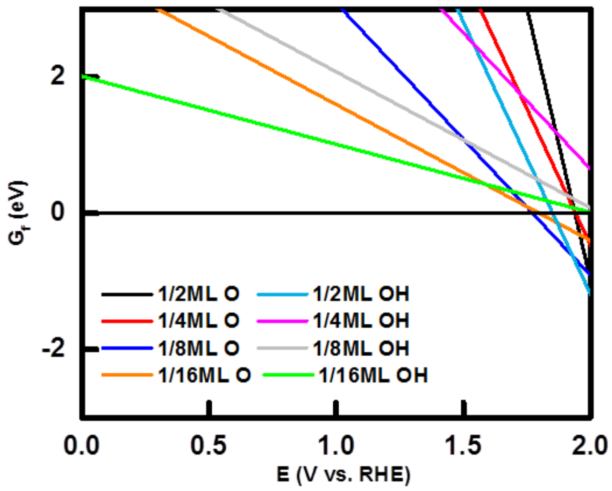

Supplementary Figure 27. Computational Pourbaix diagram of 1T-IrO_2 (0001) surface. All surface formation energies at different coverage were referenced to that of the bare surface.

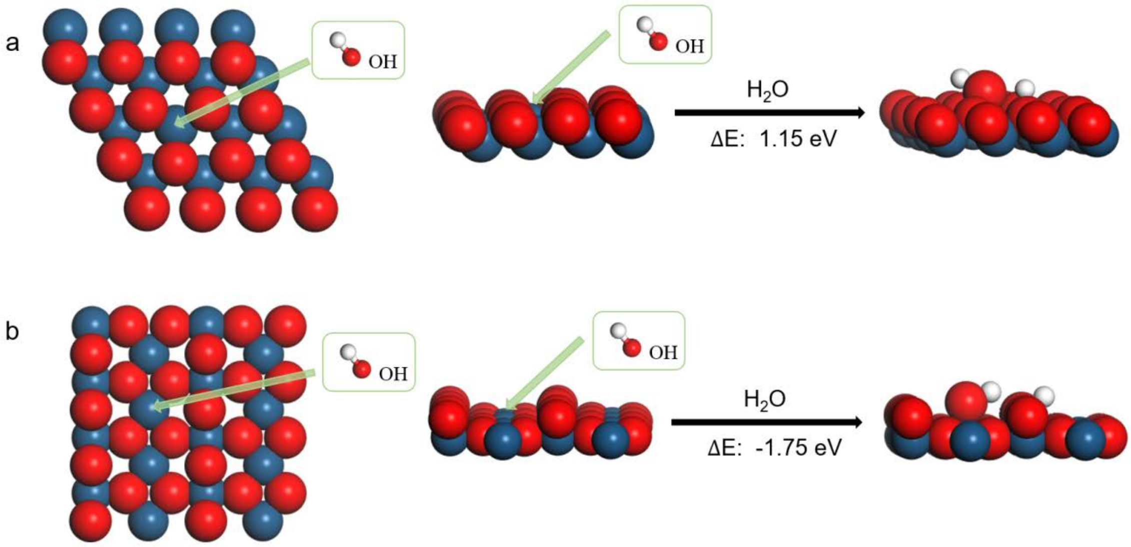

Supplementary Figure 28. The reaction energies of water splitting on a, 1T-IrO_2 and b, rutile-IrO_2. As shown, the dissociation of water is endothermic on the 1T-IrO_2, but exothermic on rutile-IrO_2. Thus, the hydroxylation of Ir on rutile-IrO_2 (110) surface should be a spontaneous process.

Supplementary Tables

Supplementary Table 1. Crystallographic information for 1T-IrO_2 and rutile-IrO_2.

Material	Crystal System	Bravais Lattice	Unit-Cell Dimensions	Space Group
1T-IrO_2	Trigona	Primitive	$a = b = 3.11$ Å, c =6.91 Å; $\alpha = \beta = 90°$, $\gamma =$ 120°	P-3m1 (164)
rutile-IrO_2	Tetragonal	Primitive	$a = b = 4.505$ Å, $c =$ 3.158 Å; $\alpha = \beta = \gamma = 90°$	$P4_2$/mnm (136)

Supplementary Table 2. The calculation of Zn^{2+} ion based area on different catalysts.

Sample	Mass (mg)	$V_{Zn}{}^{2+}$ (mL)	Zn^{2+}, before (ppm)	Zn^{2+}, after (ppm)	Catalyst Area (cm^2)	Catalyst Area per Mass (cm^2 mg^{-1})
1T-IrO_2	20.0 ± 0.01	5.0 ± 0.03	80.0 ± 0.48	53.3 ± 1.07	2089.2 ± 54.32	104.5 ± 2.77
Rutile-IrO_2	20.0 ± 0.01	5.0 ± 0.03	80.0 ± 0.48	59.8 ± 1.20	1584.1 ± 41.19	79.2 ± 2.10
C-IrO_2	20.0 ± 0.01	5.0 ± 0.03	80.0 ± 0.48	58.6 ± 1.17	1673.4 ± 43.51	83.7 ± 2.22

Supplementary Table 3. The determination of specific capacitances (C_s) for different catalysts.

Sample	C_{dl} (mF cm^{-2})	Catalyst Area per Mass (cm^2 mg^{-1})	C_s (mF cm^{-2})
1T-IrO_2	138.7 ± 3.08	104.5 ± 2.77	0.88 ± 0.043
Rutile-IrO_2	38.0 ± 0.84	79.2 ± 2.10	0.32 ± 0.016
C-IrO_2	52.8 ± 1.17	83.7 ± 2.22	0.42 ± 0.021

Supplementary Table 4. The Hg underpotential deposition and BET based areas per gram of 1T-IrO_2, Rutile-IrO_2, C-IrO_2 and C-Ir/C.

Catalyst	Hg UPD Area (m^2 g^{-1})
1T-IrO_2	15.4 ± 0.23
Rutile-IrO_2	8.8 ± 0.13
C-IrO_2	11.7 ± 0.18
C-Ir/C	10.7 ± 0.16
Catalyst	BET Area (m^2 g^{-1})
1T-IrO_2	21.9 ± 0.04
Rutile-IrO_2	11.2 ± 0.02
C-IrO_2	15.9 ± 0.03
C-Ir/C	279.8 ± 0.45

Supplementary Table 5. The Hg underpotential deposition, BET and ECSA-based areas of 1T-IrO_2, Rutile-IrO_2, C-IrO_2 and C-Ir/C. The reaction condition: GCE area: 0.0707 cm^2 and catalyst loading: 0.0146 mg.

Catalyst	Hg Deposition Based Surface Area (cm^2)
1T-IrO_2	2.3 ± 0.08
Rutile-IrO_2	1.3 ± 0.04
C-IrO_2	1.7 ± 0.06
C-Ir/C	1.6 ± 0.05
Catalyst	BET Based Surface Area (cm^2)
1T-IrO_2	3.2 ± 0.07
Rutile-IrO_2	1.6 ± 0.03
C-IrO_2	2.3 ± 0.05
C-Ir/C	40.9 ± 0.83
Catalyst	Electrochemical Surface Area (ECSA) (cm^2)
1T-IrO_2	3.6 ± 0.24
Rutile-IrO_2	2.1 ± 0.14
C-IrO_2	2.7 ± 0.18
C-Ir/C	9.4 ± 0.63

Supplementary Table 6. The Hg underpotential deposition and BET-based areas of 1T-IrO_2, Rutile-IrO_2, C-IrO_2 and C-Ir/C in the scale-up measurement. The reaction condition: GCE area: 1.0 cm^2 and catalyst loading: 0.85 mg.

Catalyst	Hg Deposition Based Surface Area (cm^2)
1T-IrO_2	133.6 ± 4.65
Rutile-IrO_2	75.5 ± 2.32
C-IrO_2	98.7 ± 3.48
C-Ir/C	92.9 ± 2.90
**Catalyst**	**BET Based Surface Area (cm^2)**
1T-IrO_2	185.9 ± 4.07
Rutile-IrO_2	92.9 ± 1.74
C-IrO_2	133.6 ± 2.90
C-Ir/C	2375.6 ± 48.21

Supplementary Table 7. The OER activities of 1T-IrO_2, Rutile-IrO_2, C-IrO_2 and C-Ir/C in different electrolytes. The geometric activity, mass activity and TOF values were obtained at 1.50 V vs. RHE. The reaction condition: GCE area: 0.0707 cm^2 and catalyst loading: 0.0146 mg. The reaction condition of scale-up experiment: GCE area: 1.0 cm^2 and catalyst loading: 0.85 mg.

Catalyst	Electrolyte	Overpotential @ 10 mA cm_{geo}^{-2} (mV)	Geometric Activity (mA cm_{geo}^{-2})	Mass Activity (mA mg_{Ir}^{-1})	TOF (s_{UPD}^{-1})	TOF (s_{BET}^{-1})	TOF (s_{ECSA}^{-1})
1T-IrO_2	0.1 M $HClO_4$	197 ± 3.7	52.7 ± 0.99	296.8 ± 5.58	4.2 ± 0.22	3.0 ± 0.12	2.7 ± 0.23
Rutile-IrO_2		297 ± 5.6	5.5 ± 0.10	30.7 ± 0.58	0.93 ± 0.049	0.76 ± 0.030	0.58 ± 0.050
C-IrO_2		276 ± 5.2	8.4 ± 0.16	47.4 ± 0.89	1.1 ± 0.06	0.81 ± 0.032	0.69 ± 0.060
C-Ir/C		311 ± 5.8	4.9 ± 0.09	118.4 ± 2.23	0.43 ± 0.023	-*	0.073 ± 0.0063
1T-IrO_2	0.1 M $HClO_4$ (Scale-up experiment)	-	201.7 ± 3.96	276.9 ± 5.21	3.9 ± 0.34	2.8 ± 0.21	-
Rutile-IrO_2		-	19.2 ± 0.35	26.4 ± 0.50	0.8 ± 0.07	0.65 ± 0.047	-
C-IrO_2		-	32.1 ± 0.61	44.0 ± 0.83	1.0 ± 0.09	0.75 ± 0.055	-
C-Ir/C		-	18.3 ± 0.34	107.4 ± 2.02	0.39 ± 0.034	-*	-
1T-IrO_2	0.5 M H_2SO_4	235 ± 4.4	25.2 ± 0.47	142.0 ± 2.67	2.0 ± 0.11	1.5 ± 0.06	1.3 ± 0.11
Rutile-IrO_2		319 ± 6.0	2.9 ± 0.05	16.3 ± 0.31	0.50 ± 0.026	0.40 ± 0.016	0.31 ± 0.027
C-IrO_2		302 ± 5.7	4.7 ± 0.09	26.5 ± 0.50	0.61 ±	0.45 ±	0.39 ±

					0.032	0.018	0.034
C-Ir/C		340 ± 6.4	1.5 ± 0.03	36.2 ± 0.68	0.13 ± 0.007	-*	0.022 ± 0.0019

*Since the surface area of C-Ir/C cannot be accurately determined by using BET due to its carbon support, the related BET based TOF was not calculated.

Supplementary Table 8. The comparison of activity performances of 1T-IrO_2 and the reported electrocatalysts in acidic OER.

Catalyst	Substrate	Electrolyte	Catalyst Loading (mg cm^{-2})	Overpotential @ 10 mA cm_{geo}^{-2} (mV)	TOF @ 1.50 V_{RHE}	Mass Activity @ 1.50 V_{RHE}	The Methods for Active Area/Active Sites	BET Area (m^2 g^{-1})	Ref.
1T-IrO_2	GCE	0.1 M O_2-saturated $HClO_4$	0.207 ± 0.0012	197 ± 3.7	4.2 ± 0.22 s_{UPD}^{-1} / 3.0 ± 0.12 s_{BET}^{-1}/ 2.7 ± 0.23 s_{ECSA}^{-1}	296.8 ± 5.58 mA mg_{Ir}^{-1}	Hg UPD / BET	21.9 ± 0.04	This work
1T-IrO_2	GCE	0.5 M O_2-saturated H_2SO_4	0.207 ± 0.0012	235 ± 4.4	2.0 ± 0.11 s_{UPD}^{-1} / 1.5 ± 0.06 s_{BET}^{-1}/ 1.3 ± 0.11 s_{ECSA}^{-1}	142.0 ± 2.67 mA mg_{Ir}^{-1}	Hg UPD / BET / Double-layer capacitance	21.9 ± 0.04	This work
IrO_x/$SrIrO_3$	$SrIrO_3$ film	0.5 M H_2SO_4	100 nm	270-290	-	-	-	-	1
Hole-doped $IrNiO_x$ core-shell	RDE	0.05 M H_2SO_4	0.0102 (Ir)	-	-	~169 mA mg_{Ir}^{-1}	-	-	2
La_2LiIrO_6	GCE	pH = 1 (H_2SO_4)	0.25	-	-	~13 mA mg^{-1}	BET	1.7	3
Amorphous Ir nanosheets	GCE	0.1 M $HClO_4$	0.2	255	0.06 s^{-1}	~90.1 mA mg^{-1}	Double-layer capacitance	-	4
$SrCo_{0.9}Ir_{0.1}O_{3-\delta}$	RDE	0.1 M $HClO_4$	0.255	~290-340	2.56 ± 0.15 s^{-1}	-	BET	0.175	5
6H-$SrIrO_3$	GCE	0.5 M H_2SO_4	0.90	248	-	~43.4 mA mg_{Ir}^{-1}	Double-layer	0.3	6

							capacitance / BET		
Iridium-based double perovskites	RRDE	0.1 M $HClO_4$	0.015 mg_{oxide} cm_{disk}^{-2}	~330	-	-	Pseudocapacitance measurements	-	7
Amorphous Li-IrO_x	GCE	0.5 M H_2SO_4	0.05	300	~0.10 s_{BET}^{-1}	~35 mA mg_{Ir}^{-1}	Cyclic voltammetry curves / BET	27	8
Ir-Ni oxide film	RDE	0.1 M $HClO_4$	-	~ 300 mV @ 7.2 mA cm_{geo}^{-2}	-	~95 mA mg^{-1}	-	-	9
IrO_2/GCN	RDE	0.5 M H_2SO_4	0.081	278	~0.014 s^{-1}	~85 mA mg^{-1}	Double-layer capacitance	-	10
IrO_x-Ir	RDE	0.5 M H_2SO_4	0.1333	~295	~0.35 s^{-1}	~10 mA mg_{Ir}^{-1}	The anodic oxidation peak of Ir^{III} to Ir^{IV} / BET	59	11
$IrNi_x$@IrO_x nanoparticles	RDE	0.05 M H_2SO_4	0.0102 (Ir)	~330	-	~68 mA mg_{Ir}^{-1}	-	-	12
IrCo@IrO_x-nl NDs	GCE	0.05 M H_2SO_4	0.0255	247	-	-	-	-	13
3D Ir superstructure	GCE	0.1 M $HClO_4$	0.0115 (Ir)	276	-	-	Double-layer capacitance	-	14
$IrNi_x$ rhombic dodecahedra	GCE	0.05 M H_2SO_4	0.0245 (Ir)	~315	-	-	-	-	15
IrNiCu double-layered nanoframe	RDE	0.1 M $HClO_4$	0.02 (Ir)	~300	-	120 mA mg_{Ir}^{-1}	-	-	16

Sr_2IrO_4	RDE	0.1 M $HClO_4$	0.08 (Ir)	286	-	~59 mA mg_{Ir}^{-1}	BET	2.8	17
$H_{3.6}IrO_4 \cdot 3.7H_2O$	GCE	0.1 M $HClO_4$	0.255	-	-	~60 mA mg_{Ir}^{-1}	BET	4.01	18
IrO_2 nanosheets	GCE	0.1 M $HClO_4$	0.00598	~420	-	~1.74 mA mg^{-1}	-	-	19
IrO_2 nanosheets	GCE	0.5 M H_2SO_4	7.1	~350	-	~75.3 mA mg_{Ir}^{-1}	-	-	20

*Some results were not specified in the literature and were estimated according to the data graphs.

Supplementary Table 9. The comparison of stability performances of 1T-IrO_2 and the reported electrocatalysts in acidic OER.

Catalyst	Electrolyte	Current Density ($mA\ cm_{geo}^{-2}$)	Time (h)	Ref.
1T-IrO_2	0.1 M O_2-saturated $HClO_4$	50	45	This work
1T-IrO_2	0.5 M O_2-saturated H_2SO_4	40	18	This work
1T-IrO_2	0.5 M O_2-saturated H_2SO_4 in PEM	250	126	This work
$IrO_x/SrIrO_3$	0.5 M H_2SO_4	10	30	1
La_2LiIrO_6	pH = 1 (H_2SO_4)	5	0.33	3
Amorphous Ir nanosheets	0.1 M $HClO_4$	10	8	4
$SrCo_{0.9}Ir_{0.1}O_{3-\delta}$	0.1 M $HClO_4$	10	3	5
6H-$SrIrO_3$	0.5 M H_2SO_4	10	30	6
Ba_2PrIrO_6	0.1 M $HClO_4$	10	1	7
Amorphous Li-IrO_x	0.5 M H_2SO_4	40	10	8
IrO_2/GCN	0.5 M H_2SO_4	20	~4	10
IrO_x-Ir	0.5 M H_2SO_4	2	~100	11
IrNix@IrO_x nanoparticles	0.05 M H_2SO_4	1	20	12
IrCo@IrO_x-nl NDs	0.05 M H_2SO_4	2.5	10	13
3D Ir superstructure	0.1 M $HClO_4$	2.5	8	14
Sr_2IrO_4	0.1 M $HClO_4$	10	~6	17
Ir/C	0.1 M $HClO_4$	10	~2	17
Sr_2CoIrO_6	0.1 M $HClO_4$	10	24	21
Ir-MnO_2	0.5 M H_2SO_4	10	650	22
Mesoporous Ir nanosheets	0.5 M H_2SO_4	10	8	23

Supplementary Table 10. ICP-MS results of 1T-IrO_2 under different potentials for500 s.

Potential (V vs. RHE)	1.5	1.6	1.7	1.8	1.9	2.0
Time (s)	500	500	500	500	500	500
Concentration (ppb)	0.215	0.234	0.209	0.223	0.233	0.222
n_{O2} (μmol)	72.8	81.7	110.7	131.2	147.2	153.5
s-number	1300899	1340000	2030000	2260000	2430000	2650000

Supplementary Table 11. The comparison of activity performances in PEM device of 1T-IrO_2 and the reported electrocatalysts in acidic OER.

Catalyst	Catalyst Loading (mg cm^{-2})	Temperature (°C)	Current Density (mA cm_{geo}^{-2}) at 1.50 V	Ref.
1T-IrO_2	0.85	65	253	This work
$W_{0.2}Er_{0.1}Ru_{0.7}O_{2-\delta}$	0.33	-	100 at 1.60 V	24
IrO_x-Ir	1	80	140	11
IrO_2	0.7	130	90	25
$Ir_{0.7}Ru_{0.3}O_2$ (TT)	1	80	70	26
$Ir_{0.7}Ru_{0.3}O_2$ (EC)	1	80	180	26
IrO_x	1.5	60	90	27
IrO_2	0.1	80	90	28
IrO_2	0.23	80	110	28
IrO_2	0.4	80	170	25
IrO_2	0.7	80	210	28
IrO_2/Ti	0.12	80	140	28
IrO_2/Ti	0.69	80	170	28
IrO_2	0.1	80	80	29
IrO_2	0.23	80	110	29
IrO_2	0.32	80	130	29
IrO_2	0.4	80	150	29
IrO_2	0.71	80	200	29
IrO_2	1.71	80	210	29
IrO_2	0.4 (Ir)	60	110	30
IrO_2	2.25 (Ir)	80	230	31
IrO_2	2	80	10	32
IrO_2	1.5	80	180	33
IrO_x	0.2	80	180	34

*Some results were not specified in the literature and were estimated according to the data graphs.

Supplementary Table 12. The oxygen productions of 1T-IrO_2 and C-IrO_2 obtain at different reaction times in PEM device. The flow rate of carrier gas (Ar) in PEM is 100 sccm.

Catalyst	Electrolyte	Current Density ($mA\ cm_{geo}^{-2}$)	Time (h)	Oxygen Production ($mmol\ h^{-1}$)
1T-IrO_2	0.5 M O_2-saturated H_2SO_4	250	1	2.44 ± 0.095
1T-IrO_2	0.5 M O_2-saturated H_2SO_4	250	24	2.36 ± 0.080
1T-IrO_2	0.5 M O_2-saturated H_2SO_4	250	72	2.29 ± 0.072
1T-IrO_2	0.5 M O_2-saturated H_2SO_4	250	125	2.30 ± 0.068

Supplementary Table 13. Comparison of the limiting overpotentials on rutile-IrO_2 (110) and 1T-IrO_2 (0001) by the constant charge model and the constant potential model.

Limiting Overpotential (V/RHE)	1T-IrO_2 (0001) (eV)	Rutile-IrO_2 (110) (eV)
Constant Charge Model (VASP)	0.52	0.59
Constant Potential Model (JDFTx)	0.41	0.75

Supplementary Table 14. Theoretical overpotentials based on the PBE, PBE-D3, RPBE, revPBE and PW91 functionals.

1T-IrO_2	PBE	PBE-D3	RPBE	revPBE	PW91
Overpotentials (V)	0.54	0.52	0.63	0.62	0.46

Reference

1. Seitz, L. C. et al. A highly active and stable $IrO_x/SrIrO_3$ catalyst for the oxygen evolution reaction. *Science* 353, 1011-1014 (2016).

2. Nong, H. N. et al. A unique oxygen ligand environment facilitates water oxidation in hole-doped $IrNiO_x$ core-shell electrocatalysts. *Nat. Catal.* 1, 841-851 (2018).

3. Grimaud, A. et al. Activation of surface oxygen sites on an iridium-based model catalyst for the oxygen evolution reaction. *Nat. Energy* 2, 16189 (2016).

4. Wu, G. et al. A general synthesis approach for amorphous noble metal nanosheets. *Nat. Commun.* 10, 4855 (2019).

5. Chen, Y. et al. Exceptionally active iridium evolved from a pseudocubic perovskite for oxygen evolution in acid. *Nat. Commun.* 10, 572 (2019).

6. Yang, L. et al. Efficient oxygen evolution electrocatalysis in acid by a perovskite with face-sharing IrO_6 octahedral dimers. *Nat. Commun.* 9, 5236 (2018).

7. Diaz-Morales, O. et al. Iridium-based double perovskites for efficient water oxidation in acid media. *Nat. Commun.* 7, 12363 (2016).

8. Gao, J. et al. Breaking long-range order in iridium oxide by alkali ion for efficient water oxidation. *J. Am. Chem. Soc.* 141, 3014-3023 (2019).

9. Reier, T. et al. Molecular insight in structure and activity of highly efficient, low-Ir Ir-Ni oxide catalysts for electrochemical water splitting (OER). *J. Am. Chem. Soc.* 137, 13031-13040 (2015).

10. Chen, J. et al. Low-coordinate iridium oxide confined on graphitic carbon nitride for highly efficient oxygen evolution. *Angew. Chem. Int. Ed.* 58, 12540-12544 (2019).

11. Lettenmeier, P. et al. Nanosized IrO_x-Ir catalyst with relevant activity for anodes of proton exchange membrane electrolysis produced by a cost-effective procedure. *Angew. Chem. Int. Ed.* 55, 742-746 (2016).

12. Nong, H. N. et al. Oxide-supported $IrNiO_x$ core-shell particles as efficient, cost-effective, and stable catalysts for electrochemical water splitting. *Angew. Chem. Int. Ed.* 54, 2975-2979 (2015).

13. Meng, G. et al. Strain regulation to optimize the acidic water oxidation performance of atomic-layer IrO_x. *Adv. Mater*. 31, 1903616 (2019).

14. Pi, Y., Zhang, N., Guo, S., Guo, J. & Huang, X. Ultrathin laminar Ir superstructure as highly efficient oxygen evolution electrocatalyst in broad pH range. *Nano Lett.* 16, 4424-4430 (2016).

15. Pi, Y., Shao, Q., Zhu, X. & Huang, X. Dynamic structure evolution of composition segregated iridium-nickel rhombic dodecahedra toward efficient oxygen evolution electrocatalysis. *ACS Nano* 12, 7371-7379 (2018).

16. Park, J. et al. Iridium-based multimetallic nanoframe@nanoframe structure: an efficient and robust electrocatalyst toward oxygen evolution reaction. *ACS Nano* 11, 5500-5509 (2017).

17. Strickler, A. L., Higgins, D. & Jaramillo, T. F. Crystalline strontium iridate particle catalysts for enhanced oxygen evolution in acid. *ACS Appl. Energy Mater.* 2, 5490-5498 (2019).

18. Zhang, R. et al. First example of protonation of ruddlesden-popper Sr_2IrO_4: A route to enhanced water oxidation catalysts. *Chem. Mater.* 32, 3499-3509 (2020).

19. Takimoto, D. et al. Synthesis and oxygen electrocatalysis of iridium oxide nanosheets. *Electrocatalysis* 8, 144-150 (2017).

20. Takimoto, D.; Ayato, Y.; Mochizuki, D. & Sugimoto, W. Lateral size effects of two-dimensional IrO_2 nanosheets towards the oxygen evolution reaction activity. *Electrochemistry* 85, 779-783 (2017).

21. Zhang, R. H. et al. A dissolution/precipitation equilibrium on the surface of iridium-based perovskites controls their activity as oxygen evolution reaction catalysts in acidic media. *Angew. Chem. Int. Ed*. 58, 4571-4575 (2019).

22. Shi, Z. P. et al. Confined Ir single sites with triggered lattice oxygen redox: toward boosted and sustained water oxidation catalysis. *Joule* 5, 2164-2176 (2021).

23. Jiang, B. et al. Mesoporous metallic iridium nanosheets. *J. Am. Chem. Soc*. 140, 12434-12441 (2018).

24. Hao, S. Y. et al. Dopants fixation of ruthenium for boosting acidic oxygen evolution stability and activity. *Nat. Commun.* 11, 5368 (2020).

25. Xu, J. et al. Oxygen evolution catalysts on supports with a 3-D ordered array structure and intrinsic proton conductivity for proton exchange membrane steam etectrolysis. *Energy Environ. Sci.* 7, 820-830 (2014).

26. Wang, L. et al. Highly active anode electrocatalysts derived from electrochemical leaching of Ru from metallic $Ir_{0.7}Ru_{0.3}$ for proton exchange membrane electrolyzers. *Nano Energy* 34, 385-391 (2017).

27. Siracusano, S., Van Dijk, N., Payne-Johnson, E., Baglio, V. & Arico, A. S. Nanosized IrO_x and $IrRuO_x$ electrocatalysts for the O_2 evolution reaction in PEM water electrolysers. *Appl. Catal. B Environ.* 164, 488-495 (2015).

28. Rozain, C., Mayousse, E., Guillet, N. & Millet, P. Influence of iridium oxide loadings on the performance of PEM water electrolysis cells: Part II - Advanced oxygen electrodes. *Appl. Catal. B Environ.* 182, 123-131 (2016).

29. Rozain, C., Mayousse, E., Guillet, N. & Millet, P. Influence of iridium oxide loadings on the performance of PEM water electrolysis cells: Part I-Pure IrO_2-based anodes. *Appl. Catal. B Environ.* 182, 153-160 (2016).

30. Siracusano, S. et al. The influence of iridium chemical oxidation state on the performance and durability of oxygen evolution catalysts in PEM electrolysis. *J. Power Sources* 366, 105-144 (2017).

31. Rakousky, C. et al. An analysis of degradation phenomena in polymer electrolyte membrane water electrolysis. *J. Power Sources* 326, 120-128 (2016).

32. Lettenmeier, P. et al. Durable membrane electrode assemblies for proton exchange membrane electrolyzer systems operating at high current densities. *Electrochim. Acta* 210, 502-511 (2016).

33. Cheng, J., Zhang, H., Chen, G. & Zhang, Y. Study of $Ir_xRu_{1-x}O_2$ oxides as anodic electrocatalysts for solid polymer electrolyte water electrolysis. *Electrochim. Acta* 54, 6250-6256 (2009).

34. Slavcheva, E. et al. Sputtered iridium oxide films as electrocatalysts for water splitting via PEM electrolysis. *Electrochim. Acta* 52, 3889-3894 (2007).

Supplementary Note

Supplementary Note 1. The determination of C_s values of 1T-IrO_2, Rutile-IrO_2 and C-IrO_2.

The method that determines the C_s values of 1T-IrO_2, Rutile-IrO_2 and C-IrO_2 was proposed by Savinell *et al*[35]. Cs values were calculated as followings:

$\mathrm{Cs} = \text{voltammetric charge} \div (\Delta \mathrm{V} \times Area_{catalyst})$,

1. The determination of current density-potential curves

Commercial-grade Ti wafer (0.5 cm × 0.5 cm) was used as the substrate for all electrodes. Before testing, Ti was treated according to the Reference 36. The catalysts solutions were obtained by mixing 2 mg catalysts (1T-IrO_2, Rutile-IrO_2 or C-IrO_2) in the solution of 390 μL isopropanol and 10 μL 0.5 wt % Nafion solution with sonication to form homogenous catalysts ink. 75 μL catalysts ink was dispersed on the Ti wafer with drying naturally for testing. The voltammetric charges were used to estimate the ECSAs of samples. All the electrochemical measurements were conducted on the CHI660E electrochemical workstation (Shanghai Chenhua, China). A traditional three-electrode system was used, where the Ti wafer was used as the working electrode, calomel electrode was used as the reference electrode, a Pt wire was used as the counter electrode, and 1.0 M H_2SO_4 was used as the electrolyte. A triangular voltage wave was applied at the sweep rate of 20 mV s^{-1} in the potential range of 0.05 - 1.0 V vs. SCE. The experiment current-potential curves for samples are shown in Supplementary Fig. 12.

2. The determination of Zn^{2+} surface area (based on 0.17 nm^2 per zinc ion)

20 mg samples were dispersed in the 5 mL solution of 0.5 M NH_4Cl and 0.00122 M ZnO, and stayed for 16 h. The absorbed amount of Zn^{2+} ions was determined by inductively coupled plasma source mass spectrometer (ICP-MS).

Reference

35. Kozawa, A. Ion-exchange adsorption of zinc and copper ions on silica. *J. Inorg. Nucl. Chem.* 21, 315-324 (1961).

36. Savinell, R. F., Zeller, R. L. & Adams, J. A. Electrochemically active surface-area-voltammetric charge correlations for ruthenium and iridium dioxide electrodes. *J. Electrochem. Soc.* 137, 489-494 (1990).

Supplementary Note 2. The simulated structure of 1T-IrO_2.

```
01-sub
Ir    O Ir    O
1.0
        12.6079998016         0.0000000000         0.0000000000
        -6.3039999008        10.9188481191         0.0000000000
         0.0000000000         0.0000000000        22.5000000000
    Ir    O
    32    64
Direct
     0.999432981         0.999035001         0.301517993
     0.499448001         0.999028027         0.301526010
     0.499424994         0.499018013         0.301519990
     0.250957996         0.750544012         0.301526994
     0.499558002         0.750437975         0.301562995
     0.250007987         0.499989003         0.301362991
     0.250961989         0.250562996         0.301517993
     0.750002027         0.999985993         0.301357001
     0.250005990         0.999980986         0.301367998
     0.750943005         0.250537008         0.301517010
     0.499568999         0.250425994         0.301571012
     0.750972986         0.750566006         0.301512986
     0.999454975         0.499058008         0.301515013
     0.999575019         0.250416011         0.301551998
     0.750020027         0.499987006         0.301366001
     0.999568999         0.750418007         0.301564008
     0.166988000         0.083342001         0.100000001
     0.666987002         0.083341002         0.100002997
     0.666988015         0.583343029         0.100002997
     0.416988999         0.833343029         0.100002997
     0.666987002         0.833342016         0.100002997
     0.416987985         0.583343029         0.100002997
     0.416987985         0.333341986         0.100002997
     0.916988015         0.083342001         0.100004002
     0.416987985         0.083341002         0.100002997
     0.916989028         0.333341986         0.100002997
     0.666988015         0.333339989         0.100002997
     0.916988015         0.833343029         0.100002997
     0.166989997         0.583343029         0.100002997
     0.166988000         0.333342999         0.100004002
     0.916989028         0.583342016         0.100002997
     0.166989997         0.833343029         0.100002997
     0.833234012         0.166403994         0.261480004
     0.583602011         0.416759998         0.261474997
```

1	0.833235979	0.666396022	0.261473000
2	0.666687012	0.583308995	0.341531008
3	0.916544974	0.583257973	0.341719002
4	0.666742027	0.833458006	0.341702014
5	0.583604991	0.916768014	0.261476010
6	0.833163023	0.416830987	0.261344999
7	0.666742980	0.333451986	0.341711998
8	0.916692019	0.333306998	0.343291014
9	0.583339989	0.666644990	0.263455003
10	0.916544020	0.083255999	0.341706008
11	0.333173007	0.916837990	0.261364013
12	0.666696012	0.083313003	0.341549009
13	0.166682005	0.083319999	0.341533989
14	0.083362997	0.166653007	0.263417006
15	0.166740000	0.333453000	0.341704011
16	0.083590001	0.416756988	0.261489004
17	0.166693002	0.583302975	0.341538012
18	0.083352000	0.666664004	0.263408989
19	0.166739002	0.833449006	0.341702998
20	0.583325028	0.166642994	0.263424009
21	0.083585002	0.916754007	0.261489987
22	0.333249986	0.166417003	0.261492997
23	0.416687995	0.333301008	0.343344003
24	0.333157003	0.416842014	0.261337012
25	0.416545004	0.583262026	0.341701001
26	0.333236009	0.666396976	0.261483014
27	0.416687995	0.833311975	0.343331993
28	0.916701972	0.833316028	0.343327999
29	0.416557997	0.083260998	0.341706008
30	0.833151996	0.916845977	0.261330009
31	0.000322000	0.250009000	0.059827998
32	0.750321984	0.500009000	0.059827998
33	0.000323000	0.750009000	0.059827000
34	0.833653986	0.666674972	0.140168995
35	0.083656996	0.666675985	0.140168995
36	0.833653986	0.916675985	0.140168995
37	0.750320017	0.000009000	0.059827998
38	0.000323000	0.500009000	0.059827998
39	0.833655000	0.416675001	0.140169993
40	0.083655998	0.416676015	0.140168995
41	0.750320017	0.750007987	0.059827998
42	0.083655000	0.166676000	0.140167996
43	0.500320971	0.000008000	0.059829000
44	0.833653986	0.166675001	0.140168995

0.333653986 0.166674003 0.140168995
0.250319988 0.250007987 0.059827000
0.333653986 0.416676998 0.140168995
0.250322014 0.500010014 0.059829000
0.333656013 0.666675985 0.140166998
0.250322998 0.750010014 0.059827998
0.333656013 0.916675985 0.140167996
0.750320017 0.250007004 0.059827000
0.250322998 0.000008000 0.059827998
0.500320971 0.250007004 0.059827998
0.583656013 0.416676015 0.140169993
0.500321984 0.500010014 0.059827000
0.583653986 0.666675985 0.140168995
0.500320971 0.750009000 0.059829000
0.583652973 0.916674972 0.140167996
0.083656996 0.916675985 0.140168995
0.583653986 0.166673005 0.140167996
0.000322000 0.000010000 0.059827998

02-OH
Ir O H Ir O
1.0
12.6079998016 0.0000000000 0.0000000000
-6.3039999008 10.9188481191 0.0000000000
0.0000000000 0.0000000000 22.5000000000
Ir O H
32 65 1
Direct
0.999679983 0.000086000 0.301147014
0.499563992 0.999185979 0.301317990
0.503283024 0.501080990 0.324131995
0.246854007 0.753368974 0.302534014
0.497758001 0.748111010 0.304798990
0.250046015 0.500868022 0.304334015
0.250097990 0.247976005 0.304369986
0.752996027 0.003574000 0.304006010
0.246904999 0.992281973 0.302556008
0.753021002 0.248189002 0.303988010
0.497795999 0.248442993 0.304814011
0.750389993 0.748269022 0.304401010
0.999634981 0.498266995 0.301104009
0.994222999 0.246505007 0.303671002
0.750388980 0.500871003 0.304361999

1	0.006257000	0.752494991	0.302242994
2	0.166988000	0.083342001	0.100000001
3	0.666987002	0.083341002	0.100002997
4	0.666988015	0.583343029	0.100002997
5	0.416988999	0.833343029	0.100002997
6	0.666987002	0.833342016	0.100002997
7	0.416987985	0.583343029	0.100002997
8	0.416987985	0.333341986	0.100002997
9	0.916988015	0.083342001	0.100004002
10	0.416987985	0.083341002	0.100002997
11	0.916989028	0.333341986	0.100002997
12	0.666988015	0.333341002	0.100002997
13	0.916988015	0.833343029	0.100002997
14	0.166989997	0.583343029	0.100002997
15	0.166988000	0.333342999	0.100004002
16	0.916989028	0.583342016	0.100002997
17	0.166989997	0.833343029	0.100002997
18	0.832153022	0.165426001	0.258022994
19	0.575142026	0.422338992	0.270962000
20	0.838580012	0.668673992	0.265597999
21	0.684665024	0.591674984	0.352384001
22	0.919734001	0.583829999	0.340611011
23	0.668563008	0.835762024	0.342972010
24	0.586750984	0.915789008	0.265906006
25	0.829836011	0.414097995	0.264654011
26	0.668618977	0.331579000	0.342943013
27	0.918109000	0.335427999	0.347142011
28	0.575194001	0.651673973	0.270927995
29	0.918121994	0.081436001	0.347110003
30	0.335314006	0.917053998	0.258318990
31	0.664274991	0.081541002	0.345297992
32	0.164750993	0.078336000	0.340833992
33	0.082736000	0.169373006	0.264501989
34	0.162111998	0.330451995	0.342189014
35	0.082725003	0.412093997	0.264421999
36	0.164681002	0.585143030	0.340802014
37	0.082317002	0.663603008	0.259651005
38	0.165893003	0.832316995	0.350084007
39	0.586772025	0.169752002	0.265891999
40	0.082393996	0.917455018	0.259665012
41	0.330240995	0.159834996	0.265554011
42	0.406863987	0.314260006	0.349875003
43	0.350580007	0.424674004	0.273066998
44	0.406767994	0.591364980	0.349864990

0.330188006	0.669155002	0.265536010
0.414678991	0.834092975	0.341636002
0.919731021	0.834599972	0.340660989
0.414730012	0.079389997	0.341666996
0.829850972	0.914493978	0.264705986
0.499092013	0.499112993	0.411282003
0.000322000	0.250009000	0.059827998
0.750321984	0.500009000	0.059827998
0.000323000	0.750009000	0.059827000
0.833653986	0.666674972	0.140168995
0.083656996	0.666675985	0.140168995
0.833653986	0.916675985	0.140168995
0.750320017	0.000009000	0.059827998
0.000323000	0.500009000	0.059827998
0.833655000	0.416675001	0.140169993
0.083655998	0.416676015	0.140168995
0.750320017	0.750007987	0.059827998
0.083655000	0.166676000	0.140167996
0.500320971	0.000008000	0.059829000
0.833653986	0.166675001	0.140168995
0.333653986	0.166674003	0.140168995
0.250319988	0.250007987	0.059827000
0.333653986	0.416676998	0.140168995
0.250322014	0.500010014	0.059829000
0.333656013	0.666675985	0.140166998
0.250322998	0.750010014	0.059827998
0.333656013	0.916675985	0.140167996
0.750320017	0.250007004	0.059827000
0.250322998	0.000008000	0.059827998
0.500320971	0.250007004	0.059827998
0.583656013	0.416676015	0.140169993
0.500321984	0.500010014	0.059827000
0.583653986	0.666675985	0.140168995
0.500320971	0.750009000	0.059829000
0.583652973	0.916674972	0.140167996
0.083656996	0.916675985	0.140168995
0.583653986	0.166673005	0.140167996
0.000322000	0.000010000	0.059827998
0.586049020	0.542159975	0.423011005

03-O

Ir O Ir O

```
1.0
        12.6079998016         0.0000000000         0.0000000000
        -6.3039999008        10.9188481191         0.0000000000
         0.0000000000         0.0000000000        22.5000000000
    Ir    O
    32   65
Direct
     0.999819994         0.000527000         0.300076991
     0.500406027         0.000428000         0.300574005
     0.500090003         0.500249028         0.329679012
     0.246453002         0.753798008         0.302201003
     0.497776985         0.749143004         0.304969013
     0.251291990         0.502561986         0.304749995
     0.251251012         0.249112993         0.304684997
     0.752268016         0.004653000         0.304073006
     0.246405005         0.993037999         0.302148014
     0.752237022         0.248069003         0.304123014
     0.497702986         0.249029994         0.304977000
     0.751271009         0.749117970         0.304737002
     0.999849975         0.499772012         0.300177991
     0.995797992         0.248108998         0.303905994
     0.751264989         0.502629995         0.304811001
     0.007331000         0.753886998         0.301982999
     0.166988000         0.083342001         0.100000001
     0.666987002         0.083341002         0.100002997
     0.666988015         0.583343029         0.100002997
     0.416988999         0.833343029         0.100002997
     0.666987002         0.833342016         0.100002997
     0.416987985         0.583343029         0.100002997
     0.416987985         0.333341986         0.100002997
     0.916988015         0.083342001         0.100004002
     0.416987985         0.083341002         0.100002997
     0.916989028         0.333341986         0.100002997
     0.666988015         0.333341002         0.100002997
     0.916988015         0.833343029         0.100002997
     0.166989997         0.583343029         0.100002997
     0.166988000         0.333342999         0.100004002
     0.916989028         0.583342016         0.100002997
     0.166989997         0.833343029         0.100002997
     0.833415985         0.166981995         0.258331001
     0.573481977         0.426818997         0.273443013
     0.839641988         0.670015991         0.264890999
     0.692833006         0.596731007         0.351651996
     0.921196997         0.585538030         0.340434998
```

1	0.668419003	0.836645007	0.343187988
2	0.586708009	0.916056991	0.265302986
3	0.828930020	0.413370997	0.265074998
4	0.668331981	0.332199991	0.343248993
5	0.918381989	0.336591989	0.346282005
6	0.573473990	0.647068024	0.273407996
7	0.918392003	0.082183003	0.346251994
8	0.335539013	0.917959988	0.258598000
9	0.663869023	0.082137004	0.346534014
10	0.164077997	0.079297997	0.340359986
11	0.084319003	0.171335995	0.264840990
12	0.163672000	0.332006007	0.343003005
13	0.084337004	0.413484007	0.264928997
14	0.164144993	0.585229993	0.340445995
15	0.082510002	0.664856017	0.258350015
16	0.166591004	0.833534002	0.349983007
17	0.586709023	0.171138003	0.265320003
18	0.082461998	0.918043017	0.258284986
19	0.330392987	0.160584003	0.265074998
20	0.403625011	0.307637006	0.351682007
21	0.353439003	0.426930994	0.273315996
22	0.403865010	0.596405983	0.351680994
23	0.330419987	0.670216024	0.265134990
24	0.414719999	0.835956991	0.340783000
25	0.921193004	0.836160004	0.340351999
26	0.414627999	0.079150997	0.340768993
27	0.828906000	0.916033983	0.264970988
28	0.500182986	0.500090003	0.408116013
29	0.000322000	0.250009000	0.059827998
30	0.750321984	0.500009000	0.059827998
31	0.000323000	0.750009000	0.059827000
32	0.833653986	0.666674972	0.140168995
33	0.083656996	0.666675985	0.140168995
34	0.833653986	0.916675985	0.140168995
35	0.750320017	0.000009000	0.059827998
36	0.000323000	0.500009000	0.059827998
37	0.833655000	0.416675001	0.140169993
38	0.083655998	0.416676015	0.140168995
39	0.750320017	0.750007987	0.059827998
40	0.083655000	0.166676000	0.140167996
41	0.500320971	0.000008000	0.059829000
42	0.833653986	0.166675001	0.140168995
43	0.333653986	0.166674003	0.140168995
44	0.250319988	0.250007987	0.059827000

```
0.333653986    0.416676998    0.140168995
0.250322014    0.500010014    0.059829000
0.333656013    0.666675985    0.140166998
0.250322998    0.750010014    0.059827998
0.333656013    0.916675985    0.140167996
0.750320017    0.250007004    0.059827000
0.250322998    0.000008000    0.059827998
0.500320971    0.250007004    0.059827998
0.583656013    0.416676015    0.140169993
0.500321984    0.500010014    0.059827000
0.583653986    0.666675985    0.140168995
0.500320971    0.750009000    0.059829000
0.583652973    0.916674972    0.140167996
0.083656996    0.916675985    0.140168995
0.583653986    0.166673005    0.140167996
0.000322000    0.000010000    0.059827998



04-OOH
Ir    O    H Ir    O
1.0
     12.6079998016     0.0000000000     0.0000000000
     -6.3039999008    10.9188481191     0.0000000000
      0.0000000000     0.0000000000    22.5000000000
   Ir    O    H
   32   66    1
Direct
0.999859989    0.999789000    0.301892996
0.499909014    0.000065000    0.301676989
0.498100996    0.501124024    0.320089996
0.247648001    0.753709018    0.302152008
0.498299986    0.748054981    0.304268003
0.250584990    0.501228988    0.303609014
0.250016004    0.248880997    0.304432988
0.750967026    0.003628000    0.304073006
0.245763004    0.992932022    0.302998990
0.754077017    0.248549998    0.303727001
0.498735011    0.248759001    0.304165006
0.749804020    0.748669982    0.304701000
0.998185992    0.498140991    0.301389009
0.994741976    0.249055997    0.303918004
0.750567973    0.500828028    0.303332001
0.005828000    0.754154027    0.302689999
```

1	0.166988000	0.083342001	0.100000001
2	0.666987002	0.083341002	0.100002997
3	0.666988015	0.583343029	0.100002997
4	0.416988999	0.833343029	0.100002997
5	0.666987002	0.833342016	0.100002997
6	0.416987985	0.583343029	0.100002997
7	0.416987985	0.333341986	0.100002997
8	0.916988015	0.083342001	0.100004002
9	0.416987985	0.083341002	0.100002997
10	0.916989028	0.333341986	0.100002997
11	0.666988015	0.333341002	0.100002997
12	0.916988015	0.833343029	0.100002997
13	0.166989997	0.583343029	0.100002997
14	0.166988000	0.333342999	0.100004002
15	0.916989028	0.583342016	0.100002997
16	0.166989997	0.833343029	0.100002997
17	0.832789004	0.165150002	0.258098990
18	0.575587988	0.423242986	0.270217001
19	0.838576972	0.669968009	0.265556991
20	0.680693984	0.589352012	0.349454999
21	0.919694006	0.585514009	0.340707988
22	0.667348981	0.836003006	0.343241006
23	0.584833980	0.915225983	0.265406013
24	0.829972982	0.413700014	0.263056010
25	0.669372976	0.331970006	0.342121989
26	0.917783022	0.336100996	0.348345995
27	0.578118026	0.655107975	0.268510997
28	0.917406976	0.082367003	0.345412999
29	0.335027993	0.918300986	0.259308010
30	0.664151013	0.082484998	0.346174002
31	0.165175006	0.080218002	0.342097998
32	0.081951998	0.169760004	0.266005993
33	0.162809998	0.332397997	0.342397988
34	0.083517000	0.414734006	0.264124006
35	0.164737001	0.584900022	0.339778006
36	0.081474997	0.664431989	0.261090010
37	0.166154996	0.832140982	0.349889010
38	0.587808013	0.169572994	0.265563011
39	0.082235999	0.917973995	0.258821011
40	0.329870999	0.160395995	0.265765011
41	0.409575999	0.319456011	0.347847998
42	0.344862014	0.421541989	0.268730998
43	0.408008009	0.589659989	0.350414008
44	0.330875009	0.669381022	0.265109003

1	0.415511012	0.834140003	0.341378003
2	0.918505013	0.834450006	0.342301995
3	0.413866013	0.079452001	0.341448992
4	0.829688013	0.915732980	0.265991986
5	0.494064003	0.508442998	0.412037998
6	0.554399014	0.455632001	0.439974010
7	0.000322000	0.250009000	0.059827998
8	0.750321984	0.500009000	0.059827998
9	0.000323000	0.750009000	0.059827000
10	0.833653986	0.666674972	0.140168995
11	0.083656996	0.666675985	0.140168995
12	0.833653986	0.916675985	0.140168995
13	0.750320017	0.000009000	0.059827998
14	0.000323000	0.500009000	0.059827998
15	0.833655000	0.416675001	0.140169993
16	0.083655998	0.416676015	0.140168995
17	0.750320017	0.750007987	0.059827998
18	0.083655000	0.166676000	0.140167996
19	0.500320971	0.000008000	0.059829000
20	0.833653986	0.166675001	0.140168995
21	0.333653986	0.166674003	0.140168995
22	0.250319988	0.250007987	0.059827000
23	0.333653986	0.416676998	0.140168995
24	0.250322014	0.500010014	0.059829000
25	0.333656013	0.666675985	0.140166998
26	0.250322998	0.750010014	0.059827998
27	0.333656013	0.916675985	0.140167996
28	0.750320017	0.250007004	0.059827000
29	0.250322998	0.000008000	0.059827998
30	0.500320971	0.250007004	0.059827998
31	0.583656013	0.416676015	0.140169993
32	0.500321984	0.500010014	0.059827000
33	0.583653986	0.666675985	0.140168995
34	0.500320971	0.750009000	0.059829000
35	0.583652973	0.916674972	0.140167996
36	0.083656996	0.916675985	0.140168995
37	0.583653986	0.166673005	0.140167996
38	0.000322000	0.000010000	0.059827998
39	0.641466022	0.520237982	0.435310990
